# Mental Health in Persons With Chronic Myeloid Leukemia During the SARS-CoV-2 Pandemic: The Need for Increased Access to Health Care Services

**DOI:** 10.3389/fpsyt.2021.679932

**Published:** 2021-06-08

**Authors:** Mei Bao, Sen Yang, Robert Peter Gale, Yanli Zhang, Xiaoli Liu, Huanling Zhu, Rong Liang, Bingcheng Liu, Li Zhou, Zongru Li, Xuelin Dou, Dayu Shi, Tao Wang, Li Meng, Weiming Li, Qian Jiang

**Affiliations:** ^1^Peking University People's Hospital, Peking University Institute of Hematology, National Clinical Research Center for Hematologic Disease, Beijing, China; ^2^Hematology Research Centre, Division of Experimental Medicine, Department of Medicine, Imperial College London, London, United Kingdom; ^3^Department of Hematology, Henan Provincial Tumor Hospital, Affiliated Cancer Hospital of Zhengzhou University, Zhengzhou, China; ^4^Department of Hematology, Nanfang Hospital, Southern Medical University, Guangzhou, China; ^5^Department of Hematology, West China Hospital, Sichuan University, Chengdu, China; ^6^Department of Hematology, Xijing Hospital, Air Force Medical University, Xi'an, China; ^7^State Key Laboratory of Experimental Hematology, National Clinical Research Center for Blood Diseases, Institute of Hematology, Chinese Academy of Medical Sciences, Tianjin, China; ^8^Shanghai Institute of Hematology, State Key Laboratory of Medical Genomics, National Research Center for Translational Medicine at Shanghai, Ruijin Hospital Affiliated to Shanghai Jiao Tong University School of Medicine, Shanghai, China; ^9^Department of Epidemiology and Biostatistics School of Public Health Peking University Health Science Center, Beijing, China; ^10^Department of Hematology, Tongji Hospital, Tongji Medical College, Huazhong University of Science and Technology, Wuhan, China; ^11^Department of Hematology, Tongji Medical College, Union Hospital, Huazhong University of Science and Technology, Wuhan, China

**Keywords:** SARS-CoV-2, chronic myeloid leukemia, mental health, depression, anxiety, distress

## Abstract

Mental health problems in the general population have been reported during the SARS-CoV-2 pandemic; however, there were rare data in persons with chronic myeloid leukemia (CML). Therefore, we performed a cross-sectional study on mental health evaluated using the 9-item Patient Health Questionnaire (PHQ-9; depression), the 7-item Generalized Anxiety Disorder (GAD-7; anxiety), and the 22-item Impact of Event Scale—Revised (IES-R; distress), including subscales of avoidance, intrusion, and hyper-arousal in persons with CML, non-cancer persons, and immediate family members of persons with cancer as controls (≥16 years) by an online survey. Data from 3,197 persons with CML and 7,256 controls were collected. In multivariate analyses, CML was significantly associated with moderate to severe depression (OR = 1.6; 95% Confidence Interval [CI], 1.4, 1.9; *p* < 0.001), anxiety (OR = 1.4 [1.1, 1.7]; *p* = 0.001), distress (OR = 1.3 [1.1, 1.5]; *p* < 0.001), and hyper-arousal (OR = 1.5 [1.3, 1.6]; *p* < 0.001). Moreover, delay in regular monitoring was significantly associated with depression (OR 1.3 [1.0, 1.7]; *p* = 0.024), anxiety (OR = 1.3 [1.0, 1.8]; *p* = 0.044), avoidance (OR = 1.2 [1.0, 1.4]; *p* = 0.017), and intrusion (OR = 1.2 [1.0, 1.4]; *p* = 0.057); tyrosine kinase-inhibitor dose reduction or discontinuation, depression (OR = 1.9 [1.3, 2.8]; *p* = 0.001), distress (OR = 2.0 [1.4, 2.8]; *p* < 0.001), avoidance (OR = 1.6 [1.2, 2.1]; *p* = 0.004), intrusion (OR = 1.6 [1.1, 2.1]; *p* = 0.006), and hyper-arousal (OR = 1.3 [1.0, 1.8]; *p* = 0.088). We concluded that persons with CML during the SARS-CoV-2 pandemic have worse mental health including depression, anxiety, and distress symptoms. Decreasing or stopping monitoring or dose resulted in adverse mental health consequences.

## Introduction

There are many reports of increased prevalence and severity of mental health problems including those of depression, anxiety, and distress during the severe acute respiratory syndrome coronavirus-2 (SARS-CoV-2) pandemic in the general population, medical health care workers (1–9), and persons with chronic diseases (10, 11). Several studies reported that cancer patients experienced mental health problems or worse health-related quality of life (HRQoL) (12–21), which were associated with delay in cancer care or reduced therapy intensity. However, the impact of the pandemic on the mental health of persons with leukemia is rare, and there is none in persons with chronic myeloid leukemia (CML).

The mental health of persons with CML is of concern because they require long-term tyrosine kinase-inhibitor (TKI) therapy response monitoring and TKI dose adjustments (22). Data from before the pandemic indicate that persons with CML experience more severe depression and/or anxiety to daily life challenges compared with controls (23–27). Several studies reported prevalence and/or severity of SARS-CoV-2-infection and of coronavirus infectious disease 2019 (COVID-19) in persons with CML (28–31) compared with controls. There are no data on the mental health of persons with CML during the SARS-CoV-2 pandemic. Therefore, we performed a cross-sectional survey to explore the prevalence of depression, anxiety, and distress symptoms in 3,197 subjects with CML during the pandemic. Results were compared with concurrent controls, which were non-cancer persons and immediate family members of persons with cancer including CML. We were especially interested in the effects on mental health of adjustments in the frequency of monitoring response to TKI therapy and/or dose.

## Materials and Methods

### Study Design

From April 28 to May 12, 2020, a cross-sectional online survey was done using the WeChat-based survey program Wenjuanwang. Persons diagnosed with CML, non-cancer persons, and immediate family members of persons with cancer including CML as controls (≥ 16 years) in China were invited to participate in the survey. Persons diagnosed with CML after February 2020 and those with COVID-19 were excluded. The studies involving human participants were reviewed and approved by the Ethics Committee of Peking University People's Hospital who waived the requirement for informed consent because of the outbreak of SARS-CoV-2.

### Questionnaire

The questionnaire consisted of five dimensions (Supplementary Contents 1, 2). The first dimension included a brief introduction of the survey and a question about whether having filled out the questionnaire before. The second dimension included 10 questions assessing demographics. The third included 15 questions about CML-related data such as diagnosis, therapy, and response. The fourth included 11 questions about behavior and experience at the peak of the COVID-19 pandemic in China, the end of January to end of March (32–34). The fifth included three self-reported validated measurement tools in Chinese version including the 9-item Patient Health Questionnaire (PHQ-9), the 7-item Generalized Anxiety Disorder (GAD-7), and the 22-item Impact of Event Scale—Revised (IES-R) to assess the prevalence and severity of depression, anxiety, and distress during the end of January to April. The questionnaire was the same for age and subjects with CML except for CML-related questions (the third dimension). Comorbidities were defined as coexisting diseases except CML and COVID-19.

### Assessment of Mental Health

The PHQ-9 (range, 0–27) was used to assess depression symptoms with the total scores categorized as follows: controls (0–4), mild (5–9), moderate (10–14), and severe (15–27) (35). The GAD-7 (range, 0–21) was used to assess anxiety symptoms with the total scores categorized as follows: controls (0–4), mild (5–9), moderate (10–14), and severe (15–21) (36). The IES-R (range, 0–88) was used to assess subjective distress (excessive panic and anxiety) caused by traumatic events including trauma-related distressing memories and persistent negative emotions resulting from the pandemic, which is composed of three subscales to measure the avoidance, intrusion, and hyper-arousal. The total IES-R score was categorized as follows: subclinical (0–8), mild (9–25), moderate (26–43), and severe (44–88) (37). Respondents who had the scores greater than the cutoff threshold of 10 in PHQ-9, 10 in GAD-7, and 26 in IES-R indicate moderate to severe depression, anxiety, and distress, respectively, and IES-R score ≥ 26 is associated with post-traumatic stress disorder (PTSD) symptoms (35, 38, 39).

### Statistical Analyses

Descriptive analysis results were presented as median (range) or number (percent). Categorical variables were compared with the chi-square test and continuous variables with the Mann–Whitney test. The k-means clustering method was used to cluster the three subscales of distress including avoidance, intrusion, and hyper-arousal symptoms derived from the IES-R. To identify the variables associated with the poor mental health in responses with CML and controls, covariates including age, sex, education level, marital status, household registration (rural vs. urban), comorbidity(ies), residence in Hubei province or elsewhere, cohabitation, behavior and experience (such as following pandemic information frequently, sharing feelings actively), and having an acute respiratory symptom during the pandemic were used to analyze the association with the moderate to severe depression, anxiety, and distress, as well as the presence of avoidance, intrusion, and hyper-arousal by the k-means clustering analysis. To identify the covariates associated with the poor mental health in persons with CML, CML-related data including disease phase, CML and TKI therapy duration, TKI used, TKI therapy line, treatment response, interruption or delay in disease monitoring, and interruption or dose reduction of TKI therapy were used to analyze. Covariates with *p* < 0.2 in univariate analysis were included in the multivariate binary logistic regression analyses. *P* < 0.05 was considered statistically significant. Analyses were conducted with SPSS Version 22.0 software.

## Results

From April 28 to May 12, 2020, data of 3,581 respondents with CML and 7,556 controls from 32 provinces and municipalities across China were collected. Questionnaires from subjects <16 years (*n* = 121), duplicates (*n* = 513), subjects with CML diagnosed after February 2020 (*n* = 42), and persons with COVID-19 (*n* = 8) were excluded. The updated dataset consisted of 3,197 respondents with CML and 7,256 controls.

### Respondent Covariates

Respondent covariates are shown in Table 1. Among the 3,197 respondents with CML, 1,831 (57%) were male. The median age was 43 years (range, 16–92 years); 732 (23%) had ≥1 comorbidity(ies); 2,989 (94%) were in the chronic phase at diagnosis; and 1,864 (58%) were receiving imatinib, 409 (13%) dasatinib, and 693 (22%), nilotinib. The median TKI therapy duration was 49 months (range, 1–228 months). A total of 427 (13%) reported that they had a negative cytogenetic analysis, which was defined as a complete cytogenetic response (CCyR); 1,782 (56%) had *BCR-ABL*_1_ gene levels ≤ 0.1% or a negative gene detection, which was defined as at least a major molecular response (MMR). Response to TKI therapy was unknown to 694 (22%) respondents. A total of 1,457 CML respondents (47%) had delay in regular monitoring because of fear of SARS-CoV-2-infection in the hospital or during travel (*n* = 866; 27%), travel restriction (*n* = 768; 24%), clinic or laboratory closure (*n* = 279; 9%), or other reasons (n = 29; 1%). Two hundred three (6%) respondents had TKI dose reduction or discontinuation because of the travel restriction (*n* = 147; 5%), fear of SARS-CoV-2 infection in the hospital or during travel (*n* = 108; 3%), clinic closure (*n* = 41; 1%), no access to TKI from patient assistance program (*n* = 39; 1%) or clinical trial (*n* = 10; 0.3%), or other reasons (*n* = 32; 1%).

**Table 1:** Respondents' characteristics.

	**CML**	**Controls**	***P*-value**
*N*	3,197	7,257	
Male, n (%)	1,831 (57)	3,296 (45)	<0.001
Age, y, median (range)	43 (16–92)	30 (16–89)	<0.001
Urban household registration, n (%)	1,888 (59)	4,200 (58)	0.260
Marital status, n (%)			<0.001
Unmarried	495 (16)	3,391 (47)	
Married	2,486 (78)	3,730 (51)	
Divorced or widowed	216 (7)	136 (2)	
Education, n (%)			<0.001
Junior middle school and below	1,012 (32)	403 (6)	
Senior middle school	786 (25)	610 (8)	
University and above	1,399 (44)	6,244 (86)	
Comorbidity(ies), n (%)	732 (23)	545 (8)	<0.001
Residence in Hubei province, n (%)	322 (10)	785 (11)	0.254
Living in rural area, n (%)	1,030 (32)	1,313 (18)	<0.001
Cohabitating with family or friends, n (%)	3,037 (95)	6,223 (86)	<0.001
Following pandemic information frequently, n (%)	1,422 (44)	3,554 (49)	<0.001
Sharing feelings actively, n (%)	2,542 (80)	6,028 (83)	<0.001
Exposure to someone with COVID-19, n (%)	9 (0.3)	162 (2)	<0.001
Family member with an acute respiratory symptom, n (%)	3 (0.1)	40 (1)	0.001
Having acute respiratory symptom, n (%)	396 (12)	622 (9)	<0.001
Fatigue	233 (7)	285 (4)	<0.001
Cough	117 (4)	252 (4)	0.633
Fever	68 (2)	95 (1)	0.002
Sore throat	85 (3)	186 (3)	0.777
Dyspnea	34 (1)	32 (0.4)	<0.001
Other	43 (1)	48 (1)	0.001
Suspected being infected with COVID-19	87 (3)	251 (4)	0.049
Going to the hospital	84 (3)	102 (1)	<0.001
Performing lung CT scan	65 (2)	82 (1)	<0.001
Having qRT-PCR test	26 (1)	54 (1)	0.709
Disease phase at diagnosis of CML, n (%)			
Chronic	2,989 (94)	NA	NA
Accelerated	128 (4)	NA	NA
Blast	20 (1)	NA	NA
Unknown	60 (2)	NA	NA
Interval from diagnosis to starting TKI therapy, mo, median (range)	0 (0–211)	NA	NA
CML duration, mo, median (range)	52 (4–325)	NA	NA
TKI therapy duration, mo, median (range)	49 (1–228)	NA	NA
Current treatment, n (%)			
Imatinib	1,864 (58)	NA	NA
Dasatinib	409 (13)	NA	NA
Nilotinib	693 (22)	NA	NA
Ponatinib	22 (1)	NA	NA
Flumatinib	9 (0.3)	NA	NA
Radotinib	9 (0.3)	NA	NA
HQP1351	117 (4)	NA	NA
Hydryurea and/or Interferon-a	12 (0.4)	NA	NA
Post-transplantation	8 (0.3)	NA	NA
Discontinuation of TKI therapy	43 (1)	NA	NA
Others	10 (0.3)	NA	NA
Current TKI therapy line, n (%)			
1st	2,274 (71)	NA	NA
2nd	696 (22)	NA	NA
3rd	192 (6)	NA	NA
4th	35 (1)	NA	NA
Response, n (%)			
< CCyR	294 (9)	NA	NA
≥CCyR	427 (13)	NA	NA
≥MMR	1,782 (56)	NA	NA
Unknown	694 (22)	NA	NA
Delay in regular monitoring, n (%)	1,487 (47)	NA	NA
The fear of SARS-CoV-2-infection	866 (27)	NA	NA
Travel restriction	768 (24)	NA	NA
Clinic or laboratory closure	279 (9)	NA	NA
Others	29 (1)	NA	NA
TKI dose reduction or discontinuation, n (%)	203 (6)	NA	NA
Travel restriction	147 (5)	NA	NA
The fear of SARS-CoV-2-infection	108 (3)	NA	NA
Clinic closure	41 (1)	NA	NA
No access to TKI from PAP	39 (1)	NA	NA
No access to TKI from clinical trial	10 (0.3)	NA	NA
Others	32 (1)	NA	NA

*CCyR, complete cytogenetic response; CML, chronic myeloid leukemia; HQP1351, a third-generation TKI under a clinical trial; MMR, major molecular response; mo, month(s); NA, not applicable; PAP, patient assistance program; qRT-PCR, qualitative real-time polymerase chain reaction; TKI, tyrosine kinase inhibitor; y, years*.

Compared with controls, more respondents with CML were male (*p* < 0.001), older (*p* < 0.001), and married (*p* < 0.001), had lower education level (*p* < 0.001), and had a comorbidity(ies) (*p* < 0.001). Proportions of respondents from Hubei province were comparable (*p* = 0.25). During the SARS-CoV-2 pandemic, more respondents with CML were rural residents (*p* < 0.001) and cohabited with their family members or friends (*p* < 0.001). Fewer respondents with CML followed pandemic information frequently (*p* < 0.001), shared feelings actively (*p* < 0.001), reported being exposed to someone with COVID-19 (*p* < 0.001), or had a family member with an acute respiratory symptom (*p* = 0.001). In contrast, more reported that they had an acute respiratory symptom (*p* < 0.001), fewer suspected that they were infected with SARS-CoV-2 (*p* = 0.049), more went to a hospital (*p* < 0.001), and had a lung computed tomography (CT) scan (*p* < 0.001).

### Comparison of Mental Health Between the Respondents With CML and Controls

**Figure 1:**
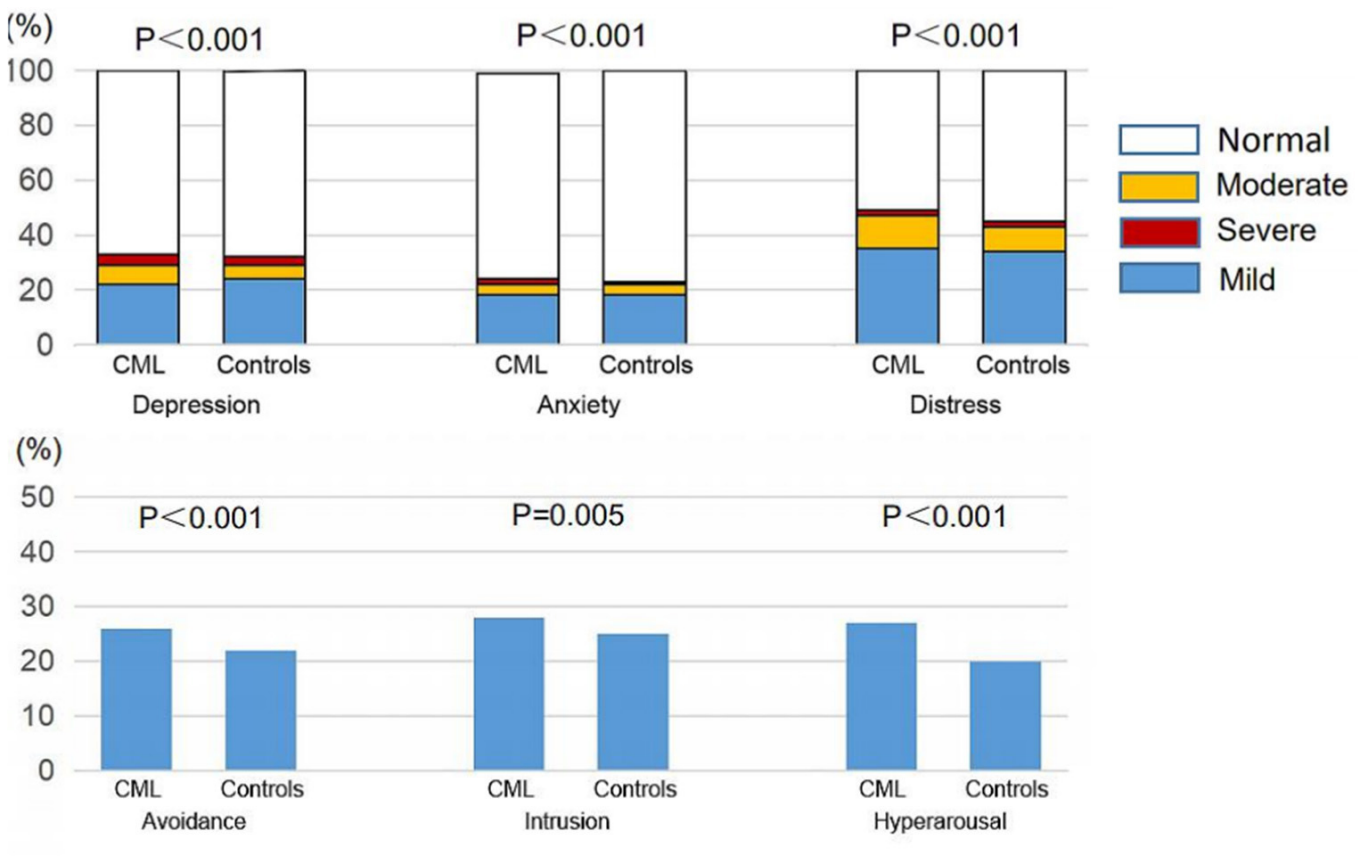
Comparison of mental health between the respondents with CML and controls.

Data from respondents with CML indicated a higher prevalence of depression (PHQ-9 score ≥ 5, 33%, 95% confidence interval [CI], [31, 33%] vs. 31% [30, 32%], *p* = 0.069), anxiety (GAD-7 score ≥ 5, 25% [24, 27%] vs. 23% [24, 27%], *p* = 0.008), and distress (IES-R score ≥ 9, 49% [47, 51%] vs. 45% [44, 46%], *p* < 0.001) and higher proportions of moderate to severe depression (PHQ-9 score ≥ 10, 11% [10, 12%] vs. 8% [7, 8%], *p* < 0.001), anxiety (GAD-7 score ≥ 10, 7% [6, 8%] vs. 5% [5, 6%], *p* < 0.001), and distress (IES-R score ≥ 26, 14% [13, 15%] vs. 10% [10, 11%], *p* < 0.001) compared with controls. There was a higher prevalence of avoidance (26% [24, 27%] vs. 22% [21, 23%], *p* < 0.001), intrusion (28% [26, 29%] vs. 25% [24, 26%], *p* = 0.005), and hyper-arousal (27% [26, 29%] vs. 20% [19, 21%], *p* < 0.001) by k-means clustering analyses (Figure 1, Table 2).

**Table 2:** Comparison of mental health of respondents with CML and controls.

	**CML**					**Controls**	***P^**1**^*-value**	***P^**2**^*-value**
	**Total**	**Subgroups with CML**						
		**Low-risk**	**Intermediate-risk**	**High-risk**	***P*-value**			
**Depression**								
≥5	1,054 (33)	1,664 (52)	1,412 (44.2)	121 (4)	<0.001	2,263 (31)	0.069	0.636
≥10	347 (11)	144 (9)	178 (13)	25 (21)	<0.001	550 (8)	<0.001	0.14
**Anxiety**								
≥5	813 (25)	373 (22)	389 (28)	51 (42)	<0.001	1,672 (23)	0.008	0.585
≥10	226 (7)	97 (6)	110 (8)	19 (16)	<0.001	364 (5)	<0.001	0.176
**Distress**								
≥9	1,558 (49)	774 (47)	705 (50)	79 (65)	<0.001	3,242 (45)	<0.001	0.174
≥26	450 (14)	202 (12)	211 (15)	37 (31)	<0.001	758 (10)	<0.001	0.044
Avoidance	818 (26)	375 (23)	390 (28)	53 (44)	<0.001	1,623 (22)	<0.001	0.88
Intrusion	889 (28)	412 (25)	420 (30)	57 (47)	<0.001	1,827 (25)	0.005	0.724
Hyper-arousal	873 (27)	427 (26)	399 (28)	47 (39)	0.004	1,641 (20)	<0.001	<0.001

*P^1^ is the comparison of mental health between the respondents with CML and controls; P^2^ is the comparison of mental health between low-risk CML cohort and controls*.

Univariate analyses results are shown in Supplementary Table 1. In multivariate analyses, having CML was significantly associated with moderate to severe depression (odds ratio [OR] = 1.6 [1.4–1.9]; *p* < 0.001), anxiety (OR = 1.4 [1.1–1.7]; *p* = 0.001), and distress (OR = 1.3 [1.1–1.5]; *p* < 0.001), as well as hyper-arousal (OR = 1.5 [1.3–1.6]; *p* < 0.001). In addition, having comorbidity(ies), following the pandemic information frequently, and having an acute respiratory symptom were significantly associated with moderate to severe depression, anxiety, distress, avoidance, intrusion, and hyper-arousal. Female sex, increasing age, unmarried, divorced, or widowed marital status, and living in Hubei province were significantly associated with ≥1 symptom of mental health described above; however, for those who were sharing feelings actively, there was no or mild depression (Table 3).

**Table 3:** Multivariate analyses of mental health of respondents with CML and controls.

	**Depression**		**Anxiety**		**Distress**		**Avoidance**		**Intrusion**		**Hyper-arousal**	
	**OR (95%Cl)**	***P*-value**	**OR (95%Cl)**	***P*-value**	**OR (95%Cl)**	***P*-value**	**OR (95%Cl)**	***P*-value**	**OR (95%Cl)**	***P*-value**	**OR (95%Cl)**	***P*-value**
Respondents with CML (ref. controls)	1.6 (1.4, 1.9)	<0.001	1.4 (1.1, 1.7)	0.001	1.3 (1.1, 1.5)	<0.001					1.5 (1.3, 1.6)	<0.001
Female (ref. male)			1.3 (1.1, 1.5)	0.008	1.2 (1.1, 1.4)	0.002	1.2 (1.1, 1.3)	<0.001	1.4 (1.3, 1.5)	<0.001	1.2 (1.1, 1.4)	<0.001
Age (years)^*^	0.8 (0.8,0.9)	<0.001	0.9 (0.8, 0.9)	0.002			1.1 (1.0, 1.1)	<0.001	1.1 (1.0, 1.1)	0.001		
Marital status		<0.001		<0.001		0.017						
Married (ref.)												
Unmarried	1.4 (1.1, 1.7)	0.002	0.9 (0.7, 1.1)	0.351	0.9 (0.8, 1.0)	0.101						
Divorced or widowed	2.3 (1.7, 3.2)	<0.001	2.1 (1.4, 3.0)	<0.001	1.4 (1.0, 1.9)	0.034						
Comorbidity(ies) (ref. none)	1.9 (1.6, 2.3)	<0.001	2.0 (1.6, 2.5)	<0.001	1.5 (1.3, 1.8)	<0.001	1.3 (1.1, 1.5)	<0.001	1.4 (1.2, 1.6)	<0.001	1.5 (1.3, 1.7)	<0.001
Residence in Hubei province (ref. elsewhere)			1.6 (1.3, 2.1)	<0.001	1.5 (1.2, 1.8)	<0.001	1.4 (1.2, 1.7)	<0.001	1.6 (1.4, 1.8)	<0.001	1.3 (1.1, 1.5)	0.004
Following pandemic information frequently (ref. none)	2.3 (2.0, 2.7)	<0.001	2.9 (2.4, 3.5)	<0.001	3.6 (3.1, 4.1)	<0.001	2.4 (2.2, 2.7)	<0.001	2.9 (2.6, 3.2)	<0.001	2.5 (2.3, 2.7)	<0.001
Sharing feelings actively (ref. none)	0.7 (0.6, 0.8)	<0.001							1.2 (1.0, 1.3)	0.033		
Having acute respiratory symptom (ref. none)	3.6 (3.0, 4.3)	<0.001	3.1 (2.5, 3.8)	<0.001	2.6 (2.2, 3.1)	<0.001	2.0 (1.8, 2.3)	<0.001	2.6 (2.3, 3.0)	<0.001	2.8 (2.4, 3.2)	<0.001

* *Linear with estimates for every 10-year increase*.

We collected data from subjects with CML on the disease phase at diagnosis, with the interval from diagnosis to starting TKI therapy, CML duration, TKI therapy duration, current therapy, the number of TKI therapy lines given, response (MMR vs. < MMR), delay in regular monitoring, and tyrosine kinase-inhibitor dose reduction or discontinuation. Univariate analysis results of CML persons and controls are shown in Supplementary Tables 2, 3; in multivariate analyses, delay in monitoring response to TKI therapy was significantly associated with reporting moderate to severe depression (OR = 1.3 [1.0–1.7]; *p* = 0.024) and anxiety (OR = 1.3 [1.0–1.8]; *p* = 0.044), avoidance (OR = 1.2 [1.0–1.4]; *p* = 0.017), and intrusion (OR = 1.2 [1.0–1.4]; *p* = 0.057). TKI therapy interruption or dose reductions were significantly associated with reporting moderate to severe depression (OR = 1.9 [1.3–2.8]; *p* = 0.001), distress (OR = 2.0 [1.4–2.8] *p* < 0.001), avoidance (OR = 1.6 [1.2–2.1]; *p* = 0.004), intrusion (OR = 1.6 [1.1–2.1]; *p* = 0.006), and hyper-arousal (OR = 1.3 [1.0–1.8]; *p* = 0.088). CML disease phase, TKI, TKI therapy duration, and therapy response were not significantly associated with mental health. Other covariates associated with mental health in subjects with CML were similar to those in controls (Tables 4, 5).

**Table 4:** Multivariate analyses of mental health of respondents with CML.

	**Depression**		**Anxiety**		**Distress**		**Avoidance**		**Intrusion**		**Hyper-arousal**	
	**OR (95%Cl)**	***P*-value**	**OR (95%Cl)**	***P*-value**	**OR (95%Cl)**	***P*-value**	**OR (95%Cl)**	***P*-value**	**OR (95%Cl)**	***P*-value**	**OR (95%Cl)**	***P*-value**
Female (ref. male)	1.3 (1.0, 1.6)	0.029	1.7 (1.3, 2.2)	<0.001	1.5 (1.2, 1.8)	<0.001	1.3 (1.1, 1.5)	0.004	1.5 (1.3, 1.7)	<0.001	1.3 (1.1, 1.6)	0.001
Age (years)^*^	0.8 (0.7, 0.9)	<0.001	0.8 (0.7, 0.9)	<0.001								
Marital status		0.061		<0.001								
Married (ref.)												
Unmarried	0.8 (0.6, 1.2)	0.341	0.5 (0.3, 0.8)	0.008								
Divorced or widowed	1.5 (1.0, 2.3)	0.041	1.9 (1.2, 3.0)	0.006								
Co-morbidity (ies) (ref. none)	1.9 (1.4, 2.4)	<0.001	1.9 (1.4, 2.6)	<0.001	1.3 (1.0, 1.7)	0.021	1.2 (1.0, 1.5)	0.071	1.4 (1.1, 1.7)	0.001	1.4 (1.2, 1.7)	<0.001
Residence in Hubei province (ref. elsewhere)							1.3 (1.0, 1.7)	0.030	1.4 (1.1, 1.8)	0.011		
Following pandemic information frequently (ref. none)	2.4 (1.9, 3.0)	<0.001	4.1 (3.0, 5.7)	<0.001	4.0 (3.2, 5.1)	<0.001	2.7 (2.3, 3.2)	<0.001	3.1 (2.6, 3.6)	<0.001	2.9 (2.4, 3.4)	<0.001
Sharing feelings actively (ref. none)									1.2 (1.0, 1.5)	0.084		
Having acute respiratory symptom (ref. none)	3.7 (2.8, 4.8)	<0.001	2.7 (1.9, 3.7)	<0.001	2.7 (2.1, 3.5)	<0.001	2.3 (1.8, 2.9)	<0.001	2.7 (2.2, 3.4)	<0.001	2.6 (2.1, 3.3)	<0.001
Delay in regular monitoring (ref. none)	1.3 (1.0, 1.7)	0.024	1.3 (1.0, 1.8)	0.044			1.2 (1.0, 1.4)	0.017	1.2 (1.0, 1.4)	0.057		
TKI dose reduction or discontinuation (ref. none)	1.9 (1.3, 2.8)	0.001			2.0 (1.4, 2.8)	<0.001	1.6 (1.2, 2.1)	0.004	1.6 (1.1, 2.1)	0.006	1.3 (1.0, 1.8)	0.088

* *Linear with estimates for every 10-year increase, TKI, tyrosine kinase-inhibitor*.

**Table 5:** Multivariate analyses of mental health of controls.

	**Depression**		**Anxiety**		**Distress**		**Avoidance**		**Intrusion**		**Hyper-arousal**	
	**OR (95%Cl)**	***P*-value**	**OR (95%Cl)**	***P*-value**	**OR (95%Cl)**	***P*-value**	**OR (95%Cl)**	***P*-value**	**OR (95%Cl)**	***P*-value**	**OR (95%Cl)**	***P*-value**
Female (ref. male)									1.4 (1.2, 1.6)	<0.001	1.2 (1.0, 1.3)	0.008
Age (years)^*^	0.8 (0.7, 1.0)	0.010					1.1 (1.0, 1.2)	0.001	1.1 (1.0, 1.1)	0.008		
Marital status		<0.001		0.029		0.018						0.096
Married (ref.)												
Unmarried	1.7 (1.3, 2.1)	<0.001	1.2 (1.0, 1.6)	0.066	0.9 (0.8, 1.1)	0.386					1.1 (1.0, 1.3)	0.112
Divorced or widowed	3.7 (2.3, 6.1)	<0.001	2.0 (1.1, 3.7)	0.028	1.9 (1.2, 3.0)	0.011					1.4 (0.9, 2.2)	0.094
Education		0.031										
Junior middle school and below (ref.)												
Senior middle school	0.6 (0.4, 1.0)	0.046										
University and above	0.6 (0.4, 0.9)	0.009										
Co-morbidity(ies) (ref. none)	2.0 (1.5, 2.6)	<0.001	2.1 (1.5, 2.8)	<0.001	1.7 (1.3, 2.1)	<0.001	1.4 (1.2, 1.7)	0.001	1.3 (1.1, 1.7)	0.004	1.7 (1.3, 2.0)	<0.001
Residence in Hubei province (ref. elsewhere)			1.6 (1.2, 2.2)	0.001	1.6 (1.3, 2.0)	<0.001	1.4 (1.2, 1.7)	<0.001	1.6 (1.4, 1.9)	<0.001	1.4 (1.3, 1.7)	0.001
Following pandemic information frequently (ref. none)	2.2 (1.8, 2.7)	<0.001	2.3 (1.8, 2.9)	<0.001	3.3 (2.8, 3.9)	<0.001	2.3 (2.0, 2.5)	<0.001	2.8 (2.5, 3.2)	<0.001	2.3 (2.0, 2.6)	<0.001
Sharing feelings actively (ref. none)	0.6 (0.5, 0.8)	<0.001										
Having acute respiratory symptom(ref. none)	3.6 (2.8, 4.5)	<0.001	3.4 (2.6, 4.4)	<0.001	2.5 (2.0, 3.1)	<0.001	1.9 (1.6, 2.3)	<0.001	2.5 (2.1, 2.9)	<0.001	2.9 (2.4, 3.4)	<0.001

* *Linear with estimates for every 10-year increase*.

### Comparison of Mental Health Between Low- and High-Risk Respondents With CML and Controls

**Figure 2:**
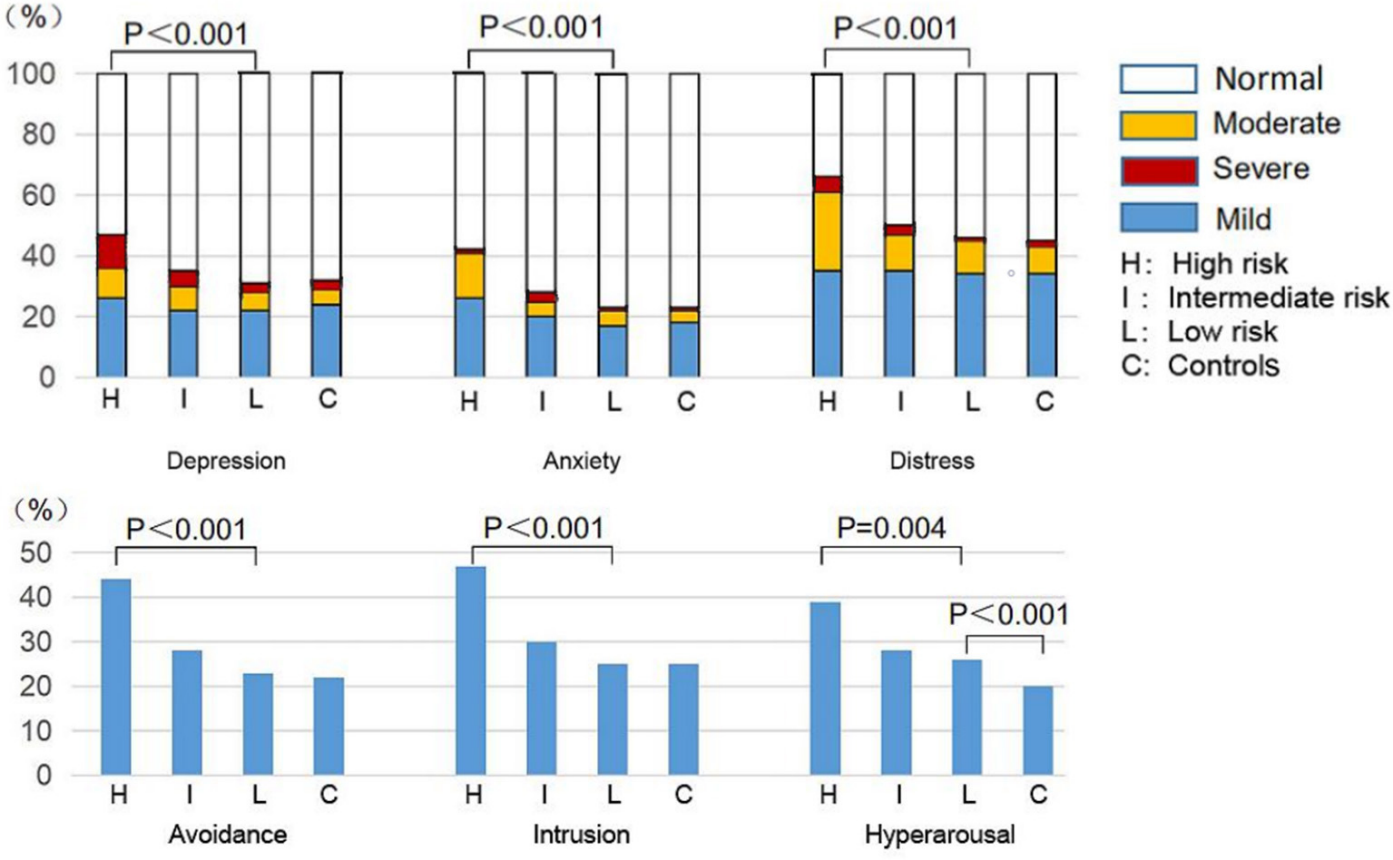
Comparison of mental health among the subgroups with CML and controls.

Next we categorized CML respondents into low-risk (no risk covariate; *n* = 1,664, 52%), intermediate-risk (1 risk; *n* = 1,412, 44%), or high-risk (2 risks, *n* = 121, 4%) cohorts based on delay in monitoring response to TKI therapy, TKI therapy interruption, or dose reduction or both during the pandemic. There were significant differences in mental health among the three CML risk cohorts or between the low-risk CML cohort and controls. The high-risk CML cohort had the highest prevalence and most severe depression (PHQ-9 score ≥ 5, 47% [38, 56%] vs. 35% [32, 37%] vs. 31% [28, 33%], *p* < 0.001 and PHQ-9 score ≥ 10, 21% [14, 29%] vs. 13% [11%, 15%] vs. 9% [7, 10%], *p* < 0.001), anxiety (GAD-7 score ≥ 5, 42% [33, 52%] vs. 28% [25, 30%] vs. 22% [20, 25%], *p* < 0.001 and GAD-7 score ≥ 10, 16% [10, 23%] vs. 8% [6, 10%] vs. 6% [5, 7%], *p* < 0.001), and distress (IES-R score ≥ 9, 65% [56, 74%] vs. 50% [47, 53%] vs. 47% [44, 49%], *p* < 0.001 and IES-R score ≥ 26, 31% [23, 40%] vs. 15% [13, 17%] vs. 12% [11, 14%], *p* < 0.001), and the highest prevalence of avoidance (44% [35%, 53%] vs. 28% [25, 30%] vs. 23% [21, 25%], *p* < 0.001), intrusion (47% [38, 56%] vs. 30% [27, 32%] vs. 25% [23, 27%], *p* < 0.001), and hyper-arousal (39% [30, 48%] vs. 28% [26, 31%] vs. 26% [24, 28%], *p* = 0.004) among the respondents with CML (Figure 2, Table 2). The low-risk CML cohort had similar mental health to controls except the higher prevalence of hyper-arousal (26 vs. 20%, *p* < 0.001). Univariate analysis results are shown in Supplementary Table 4; in multivariate analyses, subjects in the low-risk CML cohort had a significantly increased prevalence of hyper-arousal (OR = 1.4 [1.3–1.7]; *p* < 0.001) compared with controls. Other variables affecting mental health were like controls (Table 6).

**Table 6:** Multivariate analyses of mental health of respondents of low-risk group with CML and controls.

	**Depression**		**Anxiety**		**Distress**		**Avoidance**		**Intrusion**		**Hyper-arousal**	
	**OR (95%Cl)**	***P*-value**	**OR (95%Cl)**	***P*-value**	**OR (95%Cl)**	***P*-value**	**OR (95%Cl)**	***P*-value**	**OR (95%Cl)**	***P*-value**	**OR (95%Cl)**	***P*-value**
Respondents with CML (ref. controls)											1.4 (1.3, 1.7)	<0.001
Female (ref. male)					1.2 (1.0, 1.4)	0.021	1.2 (1.1, 1.3)	0.001	1.4 (1.3, 1.6)	<0.001	1.2 (1.1, 1.3)	0.004
Age (years)^*^	0.8 (0.7, 0.9)	<0.001					1.1 (1.0, 1.1)	0.001	1.1 (1.0, 1.1)	0.026		
Marital status		<0.001		0.005		0.004						0.066
Married (ref.)												
Unmarried	1.4 (1.1, 1.7)	0.002	1.2 (0.9, 1.4)	0.167	0.9 (0.8, 1.0)	0.157					1.1 (1.0, 1.3)	0.059
Divorced or widowed	3.0 (2.0, 4.4)	<0.001	2.1 (1.3, 3.4)	0.002	1.7 (1.2, 2.4)	0.006					1.3 (0.9, 1.8)	0.111
Education		0.006										
Junior middle school and below (ref.)												
Senior middle school	0.9 (0.6, 1.2)	0.353										
University and above	0.7 (0.5, 0.9)	0.003										
Comorbidity(ies) (ref. none)	2.0 (1.6, 2.5)	<0.001	2.0 (1.6, 2.6)	<0.001	1.7 (1.4, 2.0)	<0.001	1.3 (1.1, 1.6)	0.001	1.4 (1.2, 1.6)	<0.001	1.6 (1.3, 1.8)	<0.001
Residence in Hubei province (ref. elsewhere)	1.3 (1.0, 1.7)	0.048	1.6 (1.3, 2.2)	<0.001	1.5 (1.3, 1.9)	<0.001	1.4 (1.2, 1.7)	<0.001	1.6 (1.4, 1.9)	<0.001	1.3 (1.1, 1.6)	0.001
Following pandemic information frequently (ref. none)	2.2 (1.8, 2.6)	<0.001	2.6 (2.1, 3.2)	<0.001	3.4 (2.9, 4.0)	<0.001	2.4 (2.1, 2.6)	<0.001	2.8 (2.6, 3.2)	<0.001	2.4 (2.2, 2.7)	<0.001
Sharing feelings actively (ref. none)	0.7 (0.5, 0.8)	<0.001							1.2 (1.0, 1.3)	0.042		
Having acute respiratory symptom (ref. none)	3.6 (2.9, 4.4)	<0.001	3.0 (2.4, 3.8)	<0.001	2.5 (2.1, 3.0)	<0.001	1.9 (1.6, 2.3)	<0.001	2.6 (2.2, 3.0)	<0.001	2.8 (2.4, 3.3)	<0.001

* *Linear with estimates for every 10-year increase*.

## Discussion

The high- and intermediate-risk CML cohorts defined by harboring both or either of the two CML-related risk covariates including delay in monitoring response to TKI therapy and TKI therapy interruption or dose reduction during the pandemic had significantly higher prevalence of depression, anxiety, distress, avoidance, intrusion, and hyper-arousal compared with low-risk CML cohort harboring no-risk covariate. Even in the low-risk CML cohort, hyper-arousal was more common compared with controls. These data suggest that persons with CML are psychologically vulnerable during the SARS-CoV-2 pandemic.

Delay in monitoring response to TKI therapy, TKI therapy interruption, or dose reduction in persons with CML was associated with worse mental health in our study, consistent with the recent findings that treatment interruption, delay in cancer care, or reduced therapy intensity was associated with mental health problems and worse HRQoL in persons with cancer or lymphoma (12, 13, 16–18, 40–43). Fear of being infected with SARS-CoV-2 in the hospital or during travel as the common reason causing them not to follow the regular monitoring or cannot get TKI drugs in the hospital also reflected that they exaggerated the implementation of containment measures for avoidance of SARS-CoV-2 infection due to their psychological fragility.

In our study, having CML was significantly associated with moderate to severe depression, anxiety, distress, and hyper-arousal. Even in the low-risk CML cohort, hyper-arousal was more common compared with controls. Although fewer respondents with CML reported being exposed to someone with COVID-19 or a family member experiencing acute respiratory symptoms, more respondents reported that they experienced an acute respiratory symptom and went to a hospital and had a lung CT scan to evaluate COVID-19 compared with controls. These data suggest that persons with CML are psychologically more vulnerable with trauma-related distressing memories and persistent negative emotions resulting from the pandemic.

During the pandemic, although the likelihood of developing a COVID-19 infection in persons with CML is very low (28–31), negative impact on different aspects of CML management including TKI therapy response monitoring, TKI therapy, and enrollment in and compliance with clinical trials is also reported (29). These highlighted the importance of adequate access to health care services such as patient education of having appropriate personal self-protection equipment, establishing a safe area in the hospital or clinic, and telemedicine and mailed medicine to avoid monitoring and therapy interruptions^1^^,^^2^^,^^3^ (44– 50).

Our study has several limitations. First, it is cross-sectional. We lacked baseline pre-pandemic data so we cannot be certain that the observed changes in mental health were related to the pandemic. Second, there were potential selection biases in the respondents. It is not possible to assess the participation rate since it is unclear how many subjects received the link for the online survey. Third, it was impossible to confirm the accuracy and authenticity of the information provided by the respondents in the online survey. Last, the survey was conducted when the pandemic in China was mostly controlled, which might result in recall bias.

Our data indicate that persons with CML are more vulnerable than controls to mental health problems. This results in delay in monitoring TKI therapy response and in TKI therapy interruptions and dose reductions. The results of our study may help physicians identify vulnerable persons with CML and help them by increasing access to health care services.

## Data Availability Statement

The raw data supporting the conclusions of this article will be made available by the authors, without undue reservation.

## Ethics Statement

The studies involving human participants were reviewed and approved by the Ethics Committee of Peking University People's Hospital. Written informed consent from the participants' legal guardian/next of kin was not required to participate in this study in accordance with the national legislation and the institutional requirements.

## Author Contributions

The study was designed by QJ, WL, and LM. MB, SY, YZ, XL, HZ, RL, BL, LZ, ZL, XD, DS, LM, WL, and QJ collected the data. QJ, MB, SY, and TW analyzed the data. QJ, RG, MB, and SY helped prepare the typescript. All authors contributed to manuscript revision, read, and approved the submitted version.

## Conflict of Interest

The authors declare that the research was conducted in the absence of any commercial or financial relationships that could be construed as a potential conflict of interest.

## Footnotes

^1^https://www.hematology.org/covid-19/covid-19-and-cml

^2^https://covid19.aischannel.com/guidelines-nhs/files/clinical-guide-for-the-management-of-non-coronavirus-patients-requiring-acute-treatment-cancer

^3^https://www.who.int/news-room/q-a-detail/q-a-coronaviruses

## References

[1] Salari N Hosseinian-Far A Jalali R Vaisi-Raygani A Rasoulpoor S Mohammadi M. Prevalence of stress, anxiety, depression among the general population during the COVID-19 pandemic: a systematic review and meta-analysis. Global Health. (2020) 16:57. 10.1186/s12992-020-00589-w 32631403 PMC7338126

[2] Rajkumar RP. COVID-19 and mental health: a review of the existing literature. Asian J Psychiatry. (2020) 52:102066. 10.1016/j.ajp.2020.102066 32302935 PMC7151415

[3] Zhang WR Wang K Yin L Zhao WF Xue Q Peng M. Mental health and psychosocial problems of medical health workers during the COVID-19 epidemic in China. Psychother Psychosomat. (2020) 89:242–50. 10.1159/000507639 32272480 PMC7206349

[4] Cellini N Canale N Mioni G Costa S. Changes in sleep pattern, sense of time and digital media use during COVID-19 lockdown in Italy. J Sleep Res. (2020) 29:e13074. 10.31234/osf.io/284mr 32410272 PMC7235482

[5] Vindegaard N Benros ME. COVID-19 pandemic and mental health consequences: Systematic review of the current evidence. Brain Behav Immunity. (2020) 89:531–42. 10.1016/j.bbi.2020.05.048 32485289 PMC7260522

[6] Santabárbara J Lasheras I Lipnicki DM Bueno-Notivol J Moreno MP López-Antón R. Prevalence of anxiety in the COVID-19 pandemic: An updated meta-analysis of community-based studies. Prog Neuro-Psychopharmacol Biol Psychiatry. (2020) 109:110207. 10.1016/j.pnpbp.2020.110207 33338558 PMC7834650

[7] Ran MS Gao R Lin JX Zhang TM Chan SKW Deng XP. The impacts of COVID-19 outbreak on mental health in general population in different areas in China. Psychol Med. (2020) 10:1–10. 10.1017/S0033291720004717 33298220

[8] Rogers JP David AS. A longer look at COVID-19 and neuropsychiatric outcomes. Lancet Psychiatry. (2021). 10.1016/S2215-0366(21)00120-6 33836149 PMC8023693

[9] Yan H Ding Y Guo W. Mental health of medical staff during the coronavirus disease 2019 (COVID-19) pandemic: a systematic review and meta-analysis. Psycho Med. (2021) 83:387–96. 10.1097/PSY.0000000000000922 33818054

[10] Wańkowicz P Szylińska A Rotter I. Evaluation of mental health factors among people with systemic lupus erythematosus during the SARS-CoV-2 pandemic. J Clin Med. (2020) 9:2872. 10.3390/jcm9092872 32899470 PMC7563325

[11] Wańkowicz P Szylińska A Rotter I. The impact of the COVID-19 pandemic on psychological health and insomnia among people with chronic diseases. J Clin Med. (2021) 10:1206. 10.3390/jcm10061206 33799371 PMC7998391

[12] Wang Y Duan Z Ma Z Mao Y Li X Wilson A. Epidemiology of mental health problems among patients with cancer during COVID-19 pandemic. Transl Psychiatry. (2020) 10:263. 10.1038/s41398-020-00950-y 32737292 PMC7393344

[13] Chen G Wu Q Jiang H Zhang H Peng J Hu J. Fear of disease progression and psychological stress in cancer patients under the outbreak of COVID-19. Psycho-Oncology. (2020) 29:1395–8. 10.1002/pon.5451 32596867 PMC7361918

[14] Romito F Dellino M Loseto G Opinto G Silvestris E Cormio C. Psychological distress in outpatients with lymphoma during the COVID-19 pandemic. Front Oncol. (2020) 10:1270. 10.3389/fonc.2020.01270 32754447 PMC7365920

[15] Guven DC Sahin TK Aktepe OH Yildirim HC Aksoy S Kilickap S. Perspectives, knowledge, and fears of cancer patients about COVID-19. Front Oncol. (2020) 10:1553. 10.3389/fonc.2020.01553 33014800 PMC7493662

[16] Yang S Dong D Gu H Gale RP Ma J Huang X. Impact of stopping therapy during the SARS-CoV-2 pandemic in persons with lymphoma. J Cancer Res Clin Oncol. (2020) 147:1469–79. 10.1101/2020.09.28.20203083 33078214 PMC7571863

[17] Frey MK Ellis AE Zeligs K Chapman-Davis E Thomas C Christos PJ. Impact of the coronavirus disease 2019 pandemic on the quality of life for women with ovarian cancer. Am J Obstetr Gynecol. (2020) 223:725.e1–e9. 10.1016/j.ajog.2020.06.049 32598911 PMC7318934

[18] Juanjuan L Santa-Maria CA Hongfang F Lingcheng W Pengcheng Z Yuanbing X. Patient-reported outcomes of patients with breast cancer during the COVID-19 outbreak in the epicenter of China: a cross-sectional survey study. Clin Breast Cancer. (2020) 20:e651–62. 10.1016/j.clbc.2020.06.003 32709505 PMC7275993

[19] Ciazyńska M Pabianek M Szczepaniak K Ułańska M Skibińska M Owczarek W. Quality of life of cancer patients during coronavirus disease (COVID-19) pandemic. Psycho-Oncology. (2020) 29:1377–9. 10.1002/pon.5434 32779778 PMC7323427

[20] Mink van der Molen DR Bargon CA Batenburg MCT Gal R Young-Afat DA van Stam LE. (Ex-)breast cancer patients with (pre-existing) symptoms of anxiety and/or depression experience higher barriers to contact health care providers during the COVID-19 pandemic. Breast Cancer Res Treatment. (2021) 186:577–83. 10.1007/s10549-021-06112-y 33598879 PMC7889408

[21] Yu L Huang X Gale RP Wang H Jiang Q. Variables associated with patient-reported symptoms in persons with chronic phase chronic myeloid leukemia receiving tyrosine kinase inhibitor therapy. Medicine. (2019) 98:e18079. 10.1097/MD.0000000000018079 31770225 PMC6890299

[22] Kapoor J Agrawal N Ahmed R Sharma SK Gupta A Bhurani D. Factors influencing adherence to imatinib in Indian chronic myeloid leukemia patients: a cross-sectional study. Mediterranean J Hematol Infect Dis. (2015) 7:e2015013. 10.4084/mjhid.2015.013 25745540 PMC4344173

[23] Huang Y Wang Y Wang H Liu Z Yu X Yan J. Prevalence of mental disorders in China: a cross-sectional epidemiological study. Lancet Psychiatry. (2019) 6:211–24. 10.1016/S2215-0366(18)30511-X 30792114

[24] Phillips KM Pinilla-Ibarz J Sotomayor E Lee MR Jim HS Small BJ. Quality of life outcomes in patients with chronic myeloid leukemia treated with tyrosine kinase inhibitors: a controlled comparison. Support Care Cancer. (2013) 21:1097–103. 10.1007/s00520-012-1630-5 23179489

[25] Efficace F Breccia M Cottone F Okumura I Doro M Riccardi F. Psychological well-being and social support in chronic myeloid leukemia patients receiving lifelong targeted therapies. Support Care Cancer. (2016) 24:4887–94. 10.1007/s00520-016-3344-6 27448405

[26] Shi D Li Z Li Y Jiang Q. Variables associated with self-reported anxiety and depression symptoms in patients with chronic myeloid leukemia receiving tyrosine kinase inhibitor therapy. Leuke Lymphoma. (2021) 62:640–8. 10.1080/10428194.2020.1842397 33150806

[27] Efficace F Baccarani M Breccia M Alimena G Rosti G Cottone F. Health-related quality of life in chronic myeloid leukemia patients receiving long-term therapy with imatinib compared with the general population. Blood. (2011) 118:4554–60. 10.1182/blood-2011-04-347575 21750313

[28] Ector G Huijskens EGW Blijlevens NMA Westerweel PE. Prevalence of COVID-19 diagnosis in Dutch CML patients during the 2020 SARS-CoV2 pandemic. A prospective cohort study. Leukemia. (2020) 34:2533–5. 10.1038/s41375-020-0964-0 32641732 PMC7341464

[29] Breccia M Abruzzese E Bocchia M Bonifacio M Castagnetti F Fava C. Chronic myeloid leukemia management at the time of the COVID-19 pandemic in Italy. A campus CML survey. Leukemia. (2020) 34:2260–1. 10.1038/s41375-020-0904-z 32555369 PMC7301058

[30] Li W Wang D Guo J Yuan G Yang Z Gale RP. COVID-19 in persons with chronic myeloid leukaemia. Leukemia. (2020) 34:1799–804. 10.1038/s41375-020-0853-6 32424293 PMC7233329

[31] Rea D Mauro MJ Cortes JE Jiang Q Pagnano KB Ongondi M. COVID-19 in patients (pts) with Chronic Myeloid Leukemia (CML): results from the international CML foundation (iCMLf) CML and COVID-19 (CANDID) study. Blood. (2020) 136(Suppl. 1):46–7. 10.1182/blood-2020-140161

[32] Gao J Zheng P Jia Y Chen H Mao Y Chen S. Mental health problems and social media exposure during COVID-19 outbreak. PLoS ONE. (2020) 15:e0231924. 10.1371/journal.pone.0231924 32298385 PMC7162477

[33] Wang C Pan R Wan X Tan Y Xu L Ho CS. Immediate psychological responses and associated factors during the initial stage of the 2019 coronavirus disease (COVID-19) epidemic among the general population in China. Int J Environ Res Publ Health. (2020) 17:1729. 10.3390/ijerph17051729 PMC7084952 32155789

[34] Zhang Y Ma ZF. Impact of the COVID-19 pandemic on mental health and quality of life among local residents in liaoning province, China: a cross-sectional study. Int J Environ Res Publ Health. (2020) 17:2381. 10.3390/ijerph17072381 32244498 PMC7177660

[35] Manea L Gilbody S McMillan D. Optimal cut-off score for diagnosing depression with the Patient Health Questionnaire (PHQ-9): a meta-analysis. CMAJ. (2012) 184:E191–6. 10.1503/cmaj.110829 22184363 PMC3281183

[36] Spitzer RL Kroenke K Williams JB Löwe B. A brief measure for assessing generalized anxiety disorder: the GAD-7. Arch Internal Med. (2006) 166:1092–7. 10.1001/archinte.166.10.1092 16717171

[37] Wu KK Chan KS. The development of the Chinese version of Impact of Event Scale–Revised (CIES-R). Soc Psychiatry Psychiatr Epidemiol. (2003) 38:94–8. 10.1007/s00127-003-0611-x 12563552

[38] Lai J Ma S Wang Y Cai Z Hu J Wei N. Factors associated with mental health outcomes among health care workers exposed to coronavirus disease 2019. JAMA Network Open. (2020) 3:e203976. 10.1001/jamanetworkopen.2020.3976 32202646 PMC7090843

[39] Löwe B Decker O Müller S Brähler E Schellberg D Herzog W. Validation and standardization of the Generalized Anxiety Disorder Screener (GAD-7) in the general population. Med Care. (2008) 46:266–74. 10.1097/MLR.0b013e318160d093 18388841

[40] Chen-See S. Disruption of cancer care in Canada during COVID-19. Lancet Oncol. (2020) 21:e374. 10.1016/S1470-2045(20)30397-1 32711682 PMC7377791

[41] Gagliardi AR Yip CYY Irish J Wright FC Rubin B Ross H. The psychological burden of waiting for procedures and patient-centred strategies that could support the mental health of wait-listed patients and caregivers during the COVID-19 pandemic: A scoping review. Health Expect. (2021). 10.1111/hex.13241. [Epub ahead of print]. 33769657 PMC8235883

[42] Edge R Mazariego C Li Z Canfell K Miller A Koczwara B. Psychosocial impact of COVID-19 on cancer patients, survivors, and carers in Australia: a real-time assessment of cancer support services. Support Care Cancer. (2021) 11:1–11. 10.1007/s00520-021-06101-3 33694089 PMC7946616

[43] Klaassen Z Wallis CJD. Assessing patient risk from cancer and COVID-19: Managing patient distress. Urol Oncol. (2021) 39:243–6. 10.1016/j.urolonc.2021.01.023 33558139 PMC7834973

[44] Spicer J Chamberlain C Papa S. Provision of cancer care during the COVID-19 pandemic. Nat Rev Clin Oncol. (2020) 17:329–31. 10.1038/s41571-020-0370-6 32296166 PMC7156894

[45] Gosain R Abdou Y Singh A Rana N Puzanov I Ernstoff MS. COVID-19 and cancer: a comprehensive review. Curr Oncol Rep. (2020) 22:53. 10.1007/s11912-020-00934-7 32385672 PMC7206576

[46] Ueda M Martins R Hendrie PC McDonnell T Crews JR Wong TL. Managing cancer care during the COVID-19 pandemic: agility and collaboration toward a common goal. J Natl Comprehen Cancer Network: JNCCN. (2020) 20:1–4. 10.6004/jnccn.2020.7560 32197238

[47] Al-Shamsi HO Alhazzani W Alhuraiji A Coomes EA Chemaly RF Almuhanna M. A practical approach to the management of cancer patients during the novel coronavirus disease 2019 (COVID-19) pandemic: an international collaborative group. Oncologist. (2020) 25:e936–45. 10.1634/theoncologist.2020-0213 32243668 PMC7288661

[48] Hanna TP Evans GA Booth CM. Cancer, COVID-19 and the precautionary principle: prioritizing treatment during a global pandemic. Nat Rev Clin Oncol. (2020) 17:268–70. 10.1038/s41571-020-0362-6 32242095 PMC7117554

[49] von Lilienfeld-Toal M Vehreschild JJ Cornely O Pagano L Compagno F Hirsch HH. Frequently asked questions regarding SARS-CoV-2 in cancer patients-recommendations for clinicians caring for patients with malignant diseases. Leukemia. (2020) 34:1487–94. 10.1038/s41375-020-0832-y 32358568 PMC7194246

[50] Farah E Ali R Tope P El-Zein M Franco EL McGill Task Force On Covid-And C. A review of canadian cancer-related clinical practice guidelines and resources during the COVID-19 pandemic. Curr Oncol. (2021) 28:1020–33. 10.3390/curroncol28020100 33669102 PMC8025749

