# Supplementary material for: Mental Health in Persons With Chronic Myeloid Leukemia During the SARS-CoV-2 Pandemic: The Need for Increased Access to Health Care Services
Source: Front Psychiatry. 2021 Jun 8;12:679932. doi: 10.3389/fpsyt.2021.679932 (PMC8217438; doi:10.3389/fpsyt.2021.679932)
Supplement: Supplementary file 1 [file Table_1.DOCX]

Supplementary Material

# Supplementary Material

**1.1**

**Questionnaire on mental health of persons with chronic myeloid leukaemia during** **the SARS-CoV-2 pandemic**

**Part 1**

The SARS-CoV-2 pandemic has brought unparalleled psychological, social, and financial challenges to general population and persons with cancer including chronic myeloid leukaemia (CML). However, data of the impact of the pandemic on mental health of persons with CML are rare. Therefore, we conducted this study to assess the mental health of persons with CML (≥ 16 years) during the pandemic (January to April 2020).

Have you filled out this questionnaire before?

Yes

No

**Part 2**

1. Are you Chronic Myeloid Leukemia (CML) patient?

Yes

No

1. Date of filling the questionnaire?

_____________________________

1. Gender?

Male

Female

1. Age?

_____________________________

1. Household registration?

Urban

Rural

1. Marital status?

Married

Single/ Unmarried

Divorced

Widowhood

1. Educational ?

Primary school or below

Junior high school

Senior high school

University

Master or above

1. Habitual residence during the SARS-CoV-2 pandemic (2020.1-2020.4)?

_______City_______Province

1. Residence during the SARS-CoV-2 pandemic (2020.1-2020.4)?

Urban

Rural

1. Co-morbidity(ies)?

Yes

No

**Part 3**

1. When were you diagnosed with CML?

_________Year________Month

1. Disease phase at diagnosis?

Chronic

Accelerated

Blast

Unknown

1. When did you start the TKI-therapy (imatinib, dasatinib, nilotinib, or others)?

__________ Year ___________Month

1. Which type of front-line TKI drugs did you receive?

Glivec

Xinwei (Chinese generic imatinib)

GeNiKe ( Chinese generic imatinib)

Nuolining ( Chinese generic imatinib)

Foreign generic imatinib

Sprycel

Yinishu ( Chinese generic dasatinib)

Foreign generic dasatinb

Tasgina

Radotinib (in clinical trail)

Others

1. Current disease phase?

Chronic

Accelerated

Blast

Unknown

1. Have you switched TKI drugs？

No

Yes__________

1. Did you switch to which TKI drugs?

_________

1. Which TKI drug are you taking now?

Glivec

Xinwei (Chinese generic imatinib)

GeNiKe (Chinese generic imatinib)

Nuolining (Chinese generic imatinib)

Foreign generic imatinib

Sprycel

Yinishu ( Chinese generic dasatinib)

Foreign generic dasatinb

Tasgina

Ponatinib

Flumatinib

Radotinib (in clinical trail)

HQP1351 (in clinical trail)

Interferon-a

Hydryurea

Chemotherapy

Post-transplantation

Discontinuation of TKI-therapy

Others

1. Did you fail to visit clinic and monitor regularly during the SARS-CoV-2 pandemic (2020.1-2020.4)?

(within the first three months of TKI therapy, once every fortnight; thereafter, every three months until BCR-ABL1 ≤0.1%; after achieving BCR-ABL1 ≤0.1%, every three to six months; when switching treatment or having side effects, should increase the frequency of monitoring.)

Yes

No

1. The reason of failing to visit clinic and monitor? (If you answer yes to question 9, please continue)

Travel restriction

Fear of SARS-CoV-2-infection

Clinic closure

No access to TKI from patient assistance program

No access to TKI from clinical trial

Others__________

1. During the SARS-CoV-2 pandemic, did you have TKI dose reduction or discontinuation due to limited access to CML medication?

Yes

No

1. The reason of TKI dose reduction or discontinuation? (If you answer yes to question 11, please continue)

Travel restriction

Fear of SARS-CoV-2-infection

Clinic closure

No access to TKI from patient assistance program

No access to TKI from clinical trial

Others___________

1. Did you loss response or disease progression due to delay in regular monitoring or TKI dose reduction or discontinuation during the SARS-CoV-2 pandemic?

Yes

No

1. The last testing results of regular monitoring during the SARS-CoV-2 pandemic (before 2020.1)?

Time:_____Month_____Year

Complete blood count white blood cell count ______(× 10^9^/L), hemoglobin______g/L, platelet cell count____(× 10^9^/L)

BCR-ABL_1______% (IS)

Cytogenetic testing (detailed description)_________

1. The latest monitoring results (after 2020.1)?

Time:_____Month_____Year

Complete blood count white blood cell count ______(× 10^9^/L), hemoglobin______g/L, platelet cell count____(× 10^9^/L)

BCR-ABL_1______% (IS)

Cytogenetic testing (detailed description)_________

**Part 4**

1. During the SARS-CoV-2 pandemic, did you cohabitate with your family or friends?

Yes

No

1. Did you keep following pandemic information frequently and feel nervous if you don’t know the latest information?

Yes

No

1. Did you more actively share epidemic information that you thought useful?

Yes

No

1. During the SARS-CoV-2 pandemic, did you have the following acute respiratory symptoms?

Fatigue

Cough

Fever

Sore throat

Dyspnea

Others

No

(if you had the acute respiratory symptoms above, please continue to answer question 5-8)

1. Did you suspect that you have been infected with SARS-CoV-2?

Yes

No

1. Did you go to the hospital?

Yes

No

1. Did you perform lung CT scan?

Yes

No

1. Did you have qRT-PCR test?

Yes

No

1. Did you diagnose with SARS-CoV-2?

Yes

No

1. Did you expose to someone with SARS-CoV-2?

Yes

No

1. Did your family member have any of the above symptoms or diagnose with SARS-CoV-2?

Yes

No

**Part 5**

**PHQ-9**

1. Having little interest or pleasure in doing things

Not at all—0

Several days—1

More than half the days—2

Nearly every day—3

1. Feeling down, depressed, or hopeless

Not at all—0

Several days—1

More than half the days—2

Nearly every day—3

1. Having trouble falling or staying asleep, or sleeping too much

Not at all—0

Several days—1

More than half the days—2

Nearly every day—3

1. Feeling tired or having little energy

Not at all—0

Several days—1

More than half the days—2

Nearly every day—3

1. Having a poor appetite or overeating

Not at all—0

Several days—1

More than half the days—2

Nearly every day—3

1. Feeling bad about yourself — or that you are a failure or have let yourself or your family down

Not at all—0

Several days—1

More than half the days—2

Nearly every day—3

1. Having trouble concentrating on things, such as reading the newspaper or watching television

Not at all—0

Several days—1

More than half the days—2

Nearly every day—3

1. Moving or speaking so slowly that other people could have noticed? Or the opposite being so fidgety or restless that you have been moving around a lot more than usual

Not at all—0

Several days—1

More than half the days—2

Nearly every day—3

1. Having thoughts that you would be better off dead or of hurting yourself in some way

Not at all—0

Several days—1

More than half the days—2

Nearly every day—3

**GAD-7**

1. Feeling nervous, anxious or on edge

Not at all—0

Several days—1

More than half the days—2

Nearly every day—3

1. Not being able to stop or control worrying

Not at all—0

Several days—1

More than half the days—2

Nearly every day—3

1. Worrying too much about different things

Not at all—0

Several days—1

More than half the days—2

Nearly every day—3

1. Trouble relaxing

Not at all—0

Several days—1

More than half the days—2

Nearly every day—3

1. Being so restless that it is hard to sit still

Not at all—0

Several days—1

More than half the days—2

Nearly every day—3

1. Becoming easily annoyed or irritable

Not at all—0

Several days—1

More than half the days—2

Nearly every day—3

1. Feeling afraid as if something awful might happen

Not at all—0

Several days—1

More than half the days—2

Nearly every day—3

**IES-R**

1. Any reminder brought back feelings about it.

Not at all—0

A little bit—1

Moderately—2

Quite a bit—3

Extremely—4

1. I had trouble staying asleep.

Not at all—0

A little bit—1

Moderately—2

Quite a bit—3

Extremely—4

1. Other things kept making me think about it.

Not at all—0

A little bit—1

Moderately—2

Quite a bit—3

Extremely—4

1. I felt irritable and angry.

Not at all—0

A little bit—1

Moderately—2

Quite a bit—3

Extremely—4

1. I avoided letting myself get upset when I thought about it or was reminded of it.

Not at all—0

A little bit—1

Moderately—2

Quite a bit—3

Extremely—4

1. I thought about it when I didn’t mean to.

Not at all—0

A little bit—1

Moderately—2

Quite a bit—3

Extremely—4

1. I felt as if it hadn’t happened or wasn’t real..

Not at all—0

A little bit—1

Moderately—2

Quite a bit—3

Extremely—4

1. I stayed away from reminders about it.

Not at all—0

A little bit—1

Moderately—2

Quite a bit—3

Extremely—4

1. Images of it popped into my mind.

Not at all—0

A little bit—1

Moderately—2

Quite a bit—3

Extremely—4

1. I was jumpy and easily startled.

Not at all—0

A little bit—1

Moderately—2

Quite a bit—3

Extremely—4

1. I tried not to think about it.

Not at all—0

A little bit—1

Moderately—2

Quite a bit—3

Extremely—4

1. I was aware that I still had a lot of feelings about it, but I didn’t deal with them.

Not at all—0

A little bit—1

Moderately—2

Quite a bit—3

Extremely—4

1. My feelings about it were kind of numb.

Not at all—0

A little bit—1

Moderately—2

Quite a bit—3

Extremely—4

1. I found myself acting or feeling as though I was back at that time.

Not at all—0

A little bit—1

Moderately—2

Quite a bit—3

Extremely—4

1. I had trouble falling asleep.

Not at all—0

A little bit—1

Moderately—2

Quite a bit—3

Extremely—4

1. I had waves of strong feelings about it.

Not at all—0

A little bit—1

Moderately—2

Quite a bit—3

Extremely—4

1. I tried to remove it from my memory.

Not at all—0

A little bit—1

Moderately—2

Quite a bit—3

Extremely—4

1. I had trouble concentrating.

Not at all—0

A little bit—1

Moderately—2

Quite a bit—3

Extremely—4

1. Reminders of the event caused physical reactions such as sweating, difficulty in breathing, nausea or palpitations.

Not at all—0

A little bit—1

Moderately—2

Quite a bit—3

Extremely—4

1. I had dreams about it.

Not at all—0

A little bit—1

Moderately—2

Quite a bit—3

Extremely—4

1. I felt watchful or on-guard.

Not at all—0

A little bit—1

Moderately—2

Quite a bit—3

Extremely—4

1. I tried not to talk about it.

Not at all—0

A little bit—1

Moderately—2

Quite a bit—3

Extremely—4

**1.2**

**Questionnaire on mental health of general population during the SARS-CoV-2 pandemic**

**Part 1**

The SARS-CoV-2 pandemic has brought unparalleled psychological, social, and financial challenges to general population and patients with cancer including chronic myeloid leukaemia (CML). However, data of the impact of the pandemic on mental health of persons with CML are rare. Therefore, we conducted this study to assess the mental health of persons with CML (≥ 16 years) compared to general population during the pandemic (January to April 2020).

Have you filled out this questionnaire before?

Yes

No

**Part 2**

1. Are you cancer patient or immediate family members of persons with cancer?

Yes

No

1. Date of filling the questionnaire?

_____________________________

1. Gender?

Male

Female

1. Age?

_____________________________

1. Household registration?

Urban

Rural

1. Marital status?

Married

Single/ Unmarried

Divorced

Widowhood

1. Educational ?

Primary school or below

Junior high school

Senior high school

University

Master or above

1. Habitual residence during the SARS-CoV-2 pandemic (2020.1-2020.4)?

_______City_______Province

1. Residence during the SARS-CoV-2 pandemic (2020.1-2020.4)?

Urban

Rural

1. Co-morbidity(ies)?

Yes

No

**Part 3**

1. During the SARS-CoV-2 pandemic, did you cohabitate with your family or friends?

Yes

No

1. Did you keep following pandemic information frequently and feel nervous if you don’t know the latest information?

Yes

No

1. Did you more actively share epidemic information that you thought useful?

Yes

No

1. During the SARS-CoV-2 pandemic, did you have the following acute respiratory symptoms?

Fatigue

Cough

Fever

Sore throat

Dyspnea

Others

No

(if you had the acute respiratory symptoms above, please continue to answer question 5-8)

1. Did you suspect that you have been infected with SARS-CoV-2?

Yes

No

1. Did you go to the hospital?

Yes

No

1. Did you perform lung CT scan?

Yes

No

1. Did you have qRT-PCR test?

Yes

No

1. Did you diagnose with SARS-CoV-2?

Yes

No

1. Did you expose to someone with SARS-CoV-2?

Yes

No

1. Did your family member have any of the above symptoms or diagnose with SARS-CoV-2?

Yes

No

**Part 4**

**PHQ-9**

1. Having little interest or pleasure in doing things

Not at all—0

Several days—1

More than half the days—2

Nearly every day—3

1. Feeling down, depressed, or hopeless

Not at all—0

Several days—1

More than half the days—2

Nearly every day—3

1. Having trouble falling or staying asleep, or sleeping too much

Not at all—0

Several days—1

More than half the days—2

Nearly every day—3

1. Feeling tired or having little energy

Not at all—0

Several days—1

More than half the days—2

Nearly every day—3

1. Having a poor appetite or overeating

Not at all—0

Several days—1

More than half the days—2

Nearly every day—3

1. Feeling bad about yourself — or that you are a failure or have let yourself or your family down

Not at all—0

Several days—1

More than half the days—2

Nearly every day—3

1. Having trouble concentrating on things, such as reading the newspaper or watching television

Not at all—0

Several days—1

More than half the days—2

Nearly every day—3

1. Moving or speaking so slowly that other people could have noticed? Or the opposite being so fidgety or restless that you have been moving around a lot more than usual

Not at all—0

Several days—1

More than half the days—2

Nearly every day—3

1. Having thoughts that you would be better off dead or of hurting yourself in some way

Not at all—0

Several days—1

More than half the days—2

Nearly every day—3

**GAD-7**

1. Feeling nervous, anxious or on edge

Not at all—0

Several days—1

More than half the days—2

Nearly every day—3

1. Not being able to stop or control worrying

Not at all—0

Several days—1

More than half the days—2

Nearly every day—3

1. Worrying too much about different things

Not at all—0

Several days—1

More than half the days—2

Nearly every day—3

1. Trouble relaxing

Not at all—0

Several days—1

More than half the days—2

Nearly every day—3

1. Being so restless that it is hard to sit still

Not at all—0

Several days—1

More than half the days—2

Nearly every day—3

1. Becoming easily annoyed or irritable

Not at all—0

Several days—1

More than half the days—2

Nearly every day—3

1. Feeling afraid as if something awful might happen

Not at all—0

Several days—1

More than half the days—2

Nearly every day—3

**IES-R**

1. Any reminder brought back feelings about it.

Not at all—0

A little bit—1

Moderately—2

Quite a bit—3

Extremely—4

1. I had trouble staying asleep.

Not at all—0

A little bit—1

Moderately—2

Quite a bit—3

Extremely—4

1. Other things kept making me think about it.

Not at all—0

A little bit—1

Moderately—2

Quite a bit—3

Extremely—4

1. I felt irritable and angry.

Not at all—0

A little bit—1

Moderately—2

Quite a bit—3

Extremely—4

1. I avoided letting myself get upset when I thought about it or was reminded of it.

Not at all—0

A little bit—1

Moderately—2

Quite a bit—3

Extremely—4

1. I thought about it when I didn’t mean to.

Not at all—0

A little bit—1

Moderately—2

Quite a bit—3

Extremely—4

1. I felt as if it hadn’t happened or wasn’t real..

Not at all—0

A little bit—1

Moderately—2

Quite a bit—3

Extremely—4

1. I stayed away from reminders about it.

Not at all—0

A little bit—1

Moderately—2

Quite a bit—3

Extremely—4

1. Images of it popped into my mind.

Not at all—0

A little bit—1

Moderately—2

Quite a bit—3

Extremely—4

1. I was jumpy and easily startled.

Not at all—0

A little bit—1

Moderately—2

Quite a bit—3

Extremely—4

1. I tried not to think about it.

Not at all—0

A little bit—1

Moderately—2

Quite a bit—3

Extremely—4

1. I was aware that I still had a lot of feelings about it, but I didn’t deal with them.

Not at all—0

A little bit—1

Moderately—2

Quite a bit—3

Extremely—4

1. My feelings about it were kind of numb.

Not at all—0

A little bit—1

Moderately—2

Quite a bit—3

Extremely—4

1. I found myself acting or feeling as though I was back at that time.

Not at all—0

A little bit—1

Moderately—2

Quite a bit—3

Extremely—4

1. I had trouble falling asleep.

Not at all—0

A little bit—1

Moderately—2

Quite a bit—3

Extremely—4

1. I had waves of strong feelings about it.

Not at all—0

A little bit—1

Moderately—2

Quite a bit—3

Extremely—4

1. I tried to remove it from my memory.

Not at all—0

A little bit—1

Moderately—2

Quite a bit—3

Extremely—4

1. I had trouble concentrating.

Not at all—0

A little bit—1

Moderately—2

Quite a bit—3

Extremely—4

1. Reminders of the event caused physical reactions such as sweating, difficulty in breathing, nausea or palpitations.

Not at all—0

A little bit—1

Moderately—2

Quite a bit—3

Extremely—4

1. I had dreams about it.

Not at all—0

A little bit—1

Moderately—2

Quite a bit—3

Extremely—4

1. I felt watchful or on-guard.

Not at all—0

A little bit—1

Moderately—2

Quite a bit—3

Extremely—4

1. I tried not to talk about it.

Not at all—0

A little bit—1

Moderately—2

Quite a bit—3

Extremely—4

# Supplementary Figures and Tables

**Supplementary Table 1** Univariate analyses of mental health of respondents with CML and controls

|  | Depression | | Anxiety | | Distress | | Avoidance | | Intrusion | | Hyper-arousal | |
| --- | --- | --- | --- | --- | --- | --- | --- | --- | --- | --- | --- | --- |
|  | n (%) | *P* value | n (%) | *P* value | n (%) | *P* value | n (%) | *P* value | n (%) | P value | n (%) | P value |
| N | 897 (9) |  | 590 (6) |  | 1208 (12) |  | 2441 (23) |  | 2716 (26) |  | 2334 (22) |  |
| Group |  | <0.001 |  | <0.001 |  | <0.001 |  | <0.001 |  | 0.005 |  | <0.001 |
| CML | 347 (11) |  | 226 (7) |  | 450 (14) |  | 818 (26) |  | 889 (28) |  | 873 (27) |  |
| Controls | 550 (8) |  | 364 (5) |  | 758 (10) |  | 1623 (22) |  | 1827 (25) |  | 1461 (20) |  |
| Sex |  | 0.267 |  | 0.025 |  | 0.054 |  | 0.029 |  | <0.001 |  | 0.007 |
| Male | 424 (8) |  | 263 (5) |  | 561 (11) |  | 1150 (22) |  | 1211 (24) |  | 1087 (21) |  |
| Female | 473 (9) |  | 327 (6) |  | 647 (12) |  | 1291 (24) |  | 1505 (28) |  | 1247 (23) |  |
| Age, y, median (range) | 32 (16-92) | 0.028 | 34 (16-86) | 0.086 | 36 (16-92) | <0.001 | 35 (16-92) | <0.001 | 34 (16-92) | <0.001 | 34 (16-92) | <0.001 |
| Household registration |  | 0.759 |  | 0.114 |  | 0.020 |  | 0.198 |  | 0.036 |  | 0.020 |
| Urban | 518 (9) |  | 362 (6) |  | 741 (12) |  | 1449 (24) |  | 1628 (27) |  | 1408 (23) |  |
| Rural | 379 (9) |  | 228 (5) |  | 467 (11) |  | 992 (23) |  | 1088 (25) |  | 926 (21) |  |
| Marital status |  | <0.001 |  | <0.001 |  | <0.001 |  | <0.001 |  | <0.001 |  | <0.001 |
| Unmarried | 369 (10) |  | 192 (5) |  | 361 (9) |  | 795 (21) |  | 915 (24) |  | 791 (20) |  |
| Married | 467 (8) |  | 356 (6) |  | 783 (13) |  | 1550 (25) |  | 1695 (27) |  | 1444 (23) |  |
| Divorced or widowed | 61 (17) |  | 42 (122) |  | 64 (18) |  | 96 (27) |  | 106 (30) |  | 99 (28) |  |
| Education, n (%) |  | 0.002 |  | 0.065 |  | <0.001 |  | 0.003 |  | 0.683 |  | 0.001 |
| Junior middle school and below | 151 (11) |  | 91 (6) |  | 199 (14) |  | 361 (26) |  | 381 (27) |  | 352 (25) |  |
| Senior middle school | 134 (10) |  | 92 (7) |  | 198 (14) |  | 361 (26) |  | 261 (26) |  | 346 (25) |  |
| University and above | 612 (8) |  | 407 (5) |  | 811 (11) |  | 1719 (23) |  | 1974 (26) |  | 1636 (21) |  |
| Comorbidity(ies), n (%) |  | <0.001 |  | <0.001 |  | <0.001 |  | <0.001 |  | <0.001 |  | <0.001 |
| Yes | 184 (14) |  | 133 (10) |  | 230 (18) |  | 393 (31) |  | 436 (34) |  | 416 (33) |  |
| No | 691 (8) |  | 438 (5) |  | 944 (11) |  | 1977 (22) |  | 2194 (25) |  | 1844 (21) |  |
| Residential location, n (%) |  | 0.023 |  | <0.001 |  | <0.001 |  | <0.001 |  | <0.001 |  | <0.001 |
| Hubei province | 115 (10) |  | 102 (9) |  | 187 (17) |  | 341 (31) |  | 399 (36) |  | 306 (28) |  |
| Elsewhere | 782 (8) |  | 488 (5) |  | 1021 (11) |  | 2100 (23) |  | 2317 (25) |  | 2028 (22) |  |
| Residential location, n (%) |  | 0.066 |  | 0.274 |  | 0.497 |  | 0.378 |  | 0.566 |  | 0.577 |
| Urban | 674 (8) |  | 447 (6) |  | 928 (11) |  | 1878 (23) |  | 2118 (26) |  | 1801 (22) |  |
| Rural | 223 (10) |  | 143 (6) |  | 280 (12) |  | 563 (24) |  | 598 (26) |  | 322 (23) |  |
| Cohabitating with family or friends, n (%) |  | 0.247 |  | 0.652 |  | 0.566 |  | 0.839 |  | 0.228 |  | 0.170 |
| Yes | 784 (9) |  | 526 (6) |  | 1076 (12) |  | 2165 (23) |  | 2423 (26) |  | 2086 (23) |  |
| No | 113 (10) |  | 64 (5) |  | 132 (11) |  | 276 (23) |  | 293 (25) |  | 248 (21) |  |
| Following pandemic information frequently, n (%) |  | <0.001 |  | <0.001 |  | <0.001 |  | <0.001 |  | <0.001 |  | <0.001 |
| Yes | 584 (12) |  | 429 (9) |  | 903 (18) |  | 1590 (32) |  | 1836 (37) |  | 1511 (30) |  |
| No | 313 (6) |  | 161 (3) |  | 305 (6) |  | 851 (16) |  | 880 (16) |  | 823 (15) |  |
| Sharing feelings actively, n (%) |  | 0.013 |  | 0.941 |  | <0.001 |  | <0.001 |  | <0.001 |  | 0.011 |
| Yes | 708 (8) |  | 483 (6) |  | 1037 (12) |  | 2076 (24) |  | 2340 (27) |  | 1955 (23) |  |
| No | 189 (10) |  | 107 (6) |  | 171 (9) |  | 365 (19) |  | 376 (20) |  | 379 (20) |  |
| Having acute respiratory illness, n (%) |  | <0.001 |  | <0.001 |  | <0.001 |  | <0.001 |  | <0.001 |  | <0.001 |
| Yes | 240 (24) |  | 158 (16) |  | 263 (26) |  | 399 (39) |  | 486 (48) |  | 456 (45) |  |
| No | 657 (7) |  | 432 (5) |  | 945 (10) |  | 2042 (22) |  | 2230 (24) |  | 1878 (20) |  |

**Supplementary Table 2** Univariate analyses of mental health of respondents with CML

|  | Depression | | Anxiety | | Distress | | Avoidance | | Intrusion | | Hyper-arousal | |
| --- | --- | --- | --- | --- | --- | --- | --- | --- | --- | --- | --- | --- |
|  | n (%) | *P* value | n (%) | *P* value | n (%) | *P* value | n (%) | *P* value | n (%) | *P* value | n (%) | *P* value |
| N | 347 (11) |  | 226 (7) |  | 450 (14) |  | 818 (26) |  | 889 (28) |  | 873 (27) |  |
| Sex |  | 0.012 |  | <0.001 |  | <0.001 |  | 0.002 |  | <0.001 |  | <0.001 |
| Male | 177 (10) |  | 101 (6) |  | 222 (12) |  | 430 (24) |  | 449 (25) |  | 455 (25) |  |
| Female | 170 (12) |  | 125 (9) |  | 228 (17) |  | 388 (28) |  | 440 (32) |  | 418 (31) |  |
| Age, y, median (range) | 40 (16-92) | 0.010 | 41 (18-85) | 0.155 | 44 (17-92) | 0.499 | 44 (16-92) | 0.198 | 44 (17-92) | 0.086 | 43 (17-92) | 0.767 |
| Household registration |  | 0.824 |  | 0.722 |  | 0.453 |  | 0.874 |  | 0.261 |  | 0.399 |
| Urban | 203 (11) |  | 136 (7) |  | 273 (15) |  | 485 (26) |  | 539 (29) |  | 526 (28) |  |
| Rural | 144 (11) |  | 90 (7) |  | 177 (14) |  | 333 (25) |  | 350 (27) |  | 347 (27) |  |
| Marital status, n (%) |  | 0.031 |  | <0.001 |  | 0.049 |  | 0.453 |  | 0.059 |  | 0.348 |
| Unmarried | 54 (11) |  | 23 (5) |  | 54 (11) |  | 117 (24) |  | 116 (23) |  | 122 (25) |  |
| Married | 258 (10) |  | 175 (7) |  | 359 (14) |  | 641 (26) |  | 713 (29) |  | 690 (28) |  |
| Divorced or widowed | 35 (16) |  | 28 (13) |  | 37 (17) |  | 60 (28) |  | 60 (28) |  | 61(28) |  |
| Education, n (%) |  | 0.456 |  | 0.270 |  | 0.072 |  | 0.198 |  | 0.855 |  | 0.617 |
| Junior middle school and below | 110 (11) |  | 64 (6) |  | 146 (14) |  | 270 (27) |  | 285 (28) |  | 265 (26) |  |
| Senior middle school | 94 (12) |  | 65 (8) |  | 127 (16) |  | 212 (27) |  | 222 (28) |  | 217 (28) |  |
| University and above | 143 (10) |  | 97 (7) |  | 177 (13) |  | 336 (24) |  | 382 (27) |  | 391 (28) |  |
| Comorbidity (ies), n (%) |  | <0.001 |  | <0.001 |  | 0.002 |  | 0.01 |  | <0.001 |  | <0.001 |
| Yes | 114 (16) |  | 75 (10) |  | 129 (18) |  | 214 (29) |  | 247 (34) |  | 248 (34) |  |
| No | 233 (10) |  | 151 (6) |  | 321 (13) |  | 604 (25) |  | 642 (26) |  | 625 (25) |  |
| Residential location, n (%) |  | 0.037 |  | 0.010 |  | 0.021 |  | 0.001 |  | <0.001 |  | 0.184 |
| Hubei province | 46 (14) |  | 34 (11) |  | 59 (18) |  | 108 (34) |  | 119 (37) |  | 98 (30) |  |
| Elsewhere | 301 (11) |  | 192 (7) |  | 391 (14) |  | 710 (25) |  | 770 (27) |  | 775 (27) |  |
| Residential location, n (%) |  | 0.450 |  | 0.905 |  | 0.912 |  | 0.518 |  | 0.588 |  | 0.483 |
| Urban | 229 (11) |  | 154 (7) |  | 304 (14) |  | 547 (25) |  | 609 (28) |  | 600 (28) |  |
| Rural | 118 (12) |  | 72 (7) |  | 146 (14) |  | 271 (26) |  | 280 (27) |  | 273 (27) |  |
| Cohabitating with family or friends, n (%) |  | 0.722 |  | 0.827 |  | 0.903 |  | 0.702 |  | 0.416 |  | 0.393 |
| Yes | 331 (11) |  | 214 (7) |  | 428 (14) |  | 775 (26) |  | 849 (28) |  | 834 (28) |  |
| No | 16 (10) |  | 12 (8) |  | 22 (14) |  | 43 (27) |  | 40 (25) |  | 39 (24) |  |
| Following pandemic information frequently, n (%) |  | <0.001 |  | <0.001 |  | <0.001 |  | <0.001 |  | <0.001 |  | <0.001 |
| Yes | 230 (16) |  | 174 (12) |  | 333 (23) |  | 522 (37) |  | 582 (41) |  | 557 (39) |  |
| No | 117 (7) |  | 52 (3) |  | 117 (7) |  | 296 (17) |  | 307 (17) |  | 316 (18) |  |
| Sharing feelings actively, n (%) |  | 0.661 |  | 0.281 |  | 0.056 |  | 0.008 |  | <0.001 |  | 0.008 |
| Yes | 279 (11) |  | 186 (7) |  | 373 (15) |  | 677 (27) |  | 749 (30) |  | 721 (28) |  |
| No | 68 (10) |  | 40 (6) |  | 77 (12) |  | 141 (22) |  | 140 (21) |  | 152 (23) |  |
| Having acute respiratory symptom, n (%) |  | <0.001 |  | <0.001 |  | <0.001 |  | <0.001 |  | <0.001 |  | <0.001 |
| Yes | 111 (28) |  | 65 (16) |  | 119 (30) |  | 174 (44) |  | 200 (51) |  | 195 (49) |  |
| No | 236 (8) |  | 161 (6) |  | 331 (12) |  | 644 (23) |  | 689 (25) |  | 678 (24) |  |
| Disease phase at diagnosis of CML, n (%) |  | 0.030 |  | 0.095 |  | 0.546 |  | 0.506 |  | 0.86 |  | 0.722 |
| Chronic | 322 (11) |  | 213 (7) |  | 426 (14) |  | 780 (26) |  | 847 (28) |  | 831 (27) |  |
| Advanced | 9 (17) |  | 2 (4) |  | 8 (15) |  | 17 (32) |  | 15 (28) |  | 17 (32) |  |
| Unknown | 16 (18) |  | 11 (12) |  | 16 (18) |  | 21 (24) | 0.47 | 27 (30) |  | 25 (28) |  |
| Interval from diagnosis to starting TKI-therapy, mo, median (range) | 0 (0-178) | 0.391 | 0 (0-143) | 0.897 | 0 (0-178) | 0.666 | 0 (0-178) | 0.361 | 0 (0-178) | 0.808 | 0 (0-178) | 0.597 |
| CML duration, mo, median (range) | 52 (4-283) | 0.462 | 47 (4-233) | 0.084 | 53 (4-249) | 0.692 | 54 (4-249) | 0.424 | 53 (4-316) | 0.983 | 53 (4-275) | 0.859 |
| TKI-therapy duration, mo, median (range) | 47 (4-180) | 0.104 | 46 (4-227) | 0.061 | 50 (3-228) | 0.632 | 50 (3-228) |  | 50 (3-228) | 0.754 | 49 (3-228) | 0.704 |
| Current TKI used |  | 0.878 |  | 0.112 |  | 0.714 |  | 0.245 |  | 0.362 |  | 0.518 |
| First-generation | 202 (11) |  | 143 (8) |  | 269 (14) |  | 492 (26) |  | 534 (29) |  | 519 (28) |  |
| Second-generation | 123 (11) |  | 74 (7) |  | 153 (14) |  | 265 (24) |  | 300 (27) |  | 300 (27) |  |
| Others | 22 (10) |  | 9 (4) |  | 28 (13) |  | 61 (28) |  | 55 (25) |  | 54 (24) |  |
| Current TKI-therapy line, n (%) |  | 0.100 |  | 0.398 |  | 0.456 |  | 0.738 |  | 0.624 |  | 0.515 |
| 1^st^ | 234 (10) |  | 165 (7) |  | 309 (14) |  | 575 (25) |  | 626 (28) |  | 608 (27) |  |
| 2^nd^ | 91 (13) |  | 50 (7) |  | 107 (15) |  | 186 (27) |  | 203 (29) |  | 201 (29) |  |
| 3^rd^ or 4^th^ | 22 (10) |  | 11 (5) |  | 34 (15) |  | 57 (25) |  | 60 (26) |  | 64 (28) |  |
| Response, n (%) |  | 0.314 |  | 0.375 |  | 0.358 |  | 0.303 |  | 0.139 |  | 0.523 |
| <CCyR | 36 (12) |  | 21 (7) |  | 45 (15) |  | 83 (28) |  | 91 (31) |  | 87 (30) |  |
| ≥CCyR | 51 (12) |  | 35 (8) |  | 55 (13) |  | 108 (25) |  | 119 (28) |  | 124 (29) |  |
| ≥MMR | 177 (10) |  | 114 (6) |  | 240 (14) |  | 436 (25) |  | 469 (26) |  | 470 (26) |  |
| Unknown | 83 (12) |  | 56 (8) |  | 110 (16) |  | 191 (28) |  | 210 (30) |  | 192 (28) |  |
| Delay in regular monitoring, n (%) |  | 0.004 |  | 0.009 |  | 0.016 |  | <0.001 |  | 0.001 |  | 0.204 |
| Yes | 187 (13) |  | 124 (8) |  | 233 (16) |  | 425 (29) |  | 456 (31) |  | 422 (28) |  |
| No | 160 (9) |  | 102 (6) |  | 217 (13) |  | 393 (23) |  | 433 (25) |  | 451 (26) |  |
| TKI dose reduction or discontinuation, n (%) |  | <0.001 |  | 0.001 |  | <0.001 |  | <0.001 |  | <0.001 |  | <0.001 |
| Yes | 45 (22) |  | 26 (13) |  | 57 (28) |  | 82 (40) |  | 88 (43) |  | 77 (38) |  |
| No | 302 (10) |  | 200 (7) |  | 393 (13) |  | 736 (25) |  | 801 (27) |  | 796 (27) |  |

CCyR complete cytogenetic response, CML chronic myeloid leukemia, HQP1351 a 3^rd^ generation TKI under a clinical trial, MMR major molecular response, mo month(s), qRT-PCR qualitative real time polymerase chain reaction, TKI tyrosine kinase inhibitor, y years.

**Supplementary Table 3** Univariate analyses of mental health of controls

|  | Depression | | Anxiety | | Distress | | Avoidance | | Intrusion | | Hyper-arousal | |
| --- | --- | --- | --- | --- | --- | --- | --- | --- | --- | --- | --- | --- |
|  | n (%) | *P* value | n (%) | *P* value | n (%) | *P* value | n (%) | *P* value | n (%) | *P* value | n (%) | *P* value |
| N | 550 (8) |  | 364 (5) |  | 758 (10) |  | 1623 (22) |  | 1827 (25) |  | 1461 (20) |  |
| Sex |  | 0.803 |  | 0.720 |  | 0.685 |  | 0.332 |  | <0.001 |  | 0.064 |
| Male | 247 (8) |  | 162 (5) |  | 339 (10) |  | 720 (22) |  | 762 (23) |  | 632 (19) |  |
| Female | 303 (8) |  | 202 (5) |  | 419 (11) |  | 903 (23) |  | 1065 (27) |  | 829 (21) |  |
| Age, y, median (range) | 28 (16-81) | <0.001 | 30 (16-86) | 0.604 | 31 (16-77) | <0.001 | 31 (16-83) | <0.001 | 30 (16-86) | <0.001 | 30 (16-81) | 0.001 |
| Household registration |  | 0.766 |  | 0.095 |  | 0.023 |  | 0.159 |  | 0.083 |  | 0.031 |
| Urban | 315 (8) |  | 226 (5) |  | 468 (11) |  | 964 (23) |  | 1089 (26) |  | 882 (21) |  |
| Rural | 235 (8) |  | 138 (5) |  | 290 (10) |  | 659 (22) |  | 738 (24) |  | 579 (19) |  |
| Marital status |  | <0.001 |  | 0.017 |  | <0.001 |  | <0.001 |  | 0.002 |  | 0.063 |
| Unmarried | 315 (9) |  | 169 (5) |  | 307 (9) |  | 678 (20) |  | 799 (24) |  | 669 (20) |  |
| Married | 209 (6) |  | 181 (5) |  | 424 (11) |  | 909 (24) |  | 982 (26) |  | 754 (20) |  |
| Divorced or widowed | 26 (19) |  | 14 (10) |  | 27 (20) |  | 36 (27) |  | 46 (34) |  | 38 (28) |  |
| Education, n (%) |  | 0.090 |  | 0.237 |  | 0.098 |  | 0.434 |  | 0.275 |  | 0.587 |
| Junior middle school and below | 41 (10) |  | 27 (7) |  | 53 (13) |  | 91 (23) |  | 96 (24) |  | 87 (22) |  |
| Senior middle school | 40 (7) |  | 27 (4) |  | 71 (12) |  | 149 (24) |  | 139 (23) |  | 129 (21) |  |
| University and above | 469 (8) |  | 310 (5) |  | 634 (10) |  | 1383 (22) |  | 1592 (26) |  | 1245 (20) |  |
| Comorbidity(ies), n (%) |  | <0.001 |  | <0.001 |  | <0.001 |  | <0.001 |  | <0.001 |  | <0.001 |
| Yes | 70 (13) |  | 58 (5) |  | 101 (19) |  | 179 (33) |  | 189 (35) |  | 168 (31) |  |
| No | 458 (7) |  | 287 (5) |  | 623 (10) |  | 1373 (22) |  | 1552 (24) |  | 1219 (19) |  |
| Residential location, n (%) |  | 0.175 |  | <0.001 |  | <0.001 |  | <0.001 |  | <0.001 |  | <0.001 |
| Hubei province | 69 (9) |  | 68 (9) |  | 128 (16) |  | 233 (30) |  | 280 (36) |  | 208 (27) |  |
| Elsewhere | 481 (7) |  | 296 (5) |  | 630 (10) |  | 1390 (22) |  | 1547 (24) |  | 1253 (19) |  |
| Residential location, n (%) |  | 0.527 |  | 0.473 |  | 0.754 |  | 0.904 |  | 0.378 |  | 0.742 |
| Urban | 445 (8) |  | 293 (5) |  | 624 (11) |  | 1331 (22) |  | 1509 (25) |  | 1201 (20) |  |
| Rural | 105 (8) |  | 71 (5) |  | 134 (10) |  | 292 (22) |  | 318 (24) |  | 260 (20) |  |
| Cohabitating with family or friends, n (%) |  | 0.018 |  | 0.983 |  | 0.826 |  | 0.888 |  | 0.571 |  | 0.944 |
| Yes | 453 (7) |  | 312 (5) |  | 648 (10) |  | 1390 (22) |  | 1574 (25) |  | 1252 (20) |  |
| No | 97 (10) |  | 52 (5) |  | 110 (11) |  | 233 (23) |  | 253 (25) |  | 209 (20) |  |
| Following pandemic information frequently, n (%) |  | <0.001 |  | <0.001 |  | <0.001 |  | <0.001 |  | <0.001 |  | <0.001 |
| Yes | 354 (10) |  | 255 (7) |  | 570 (16) |  | 1068 (30) |  | 1254 (35) |  | 954 (27) |  |
| No | 196 (5) |  | 109 (3) |  | 188 (5) |  | 555 (15) |  | 573 (16) |  | 507 (14) |  |
| Sharing feelings actively, n (%) |  | 0.001 |  | 0.443 |  | <0.001 |  | <0.001 |  | <0.001 |  | 0.111 |
| Yes | 429 (7) |  | 297 (5) |  | 664 (11) |  | 1399 (23) |  | 1591 (26) |  | 1234 (21) |  |
| No | 121 (10) |  | 67 (6) |  | 94 (8) |  | 224 (18) |  | 236 (19) |  | 227 (19) |  |
| Having acute respiratory symptom , n (%) |  | <0.001 |  | <0.001 |  | <0.001 |  | <0.001 |  | <0.001 |  | <0.001 |
| Yes | 119 (21) |  | 93 (15) |  | 144 (23) |  | 209 (38) |  | 262 (47) |  | 237 (43) |  |
| No | 431 (6) |  | 271 (4) |  | 614 (9) |  | 1414 (21) |  | 1565 (23) |  | 1224 (18) |  |

**Supplementary Table 4** Univariate analyses of mental health of respondents of low-risk group with CML and controls

|  | Depression | | Anxiety | | Distress | | Avoidance | | Intrusion | | Hyper-arousal | |
| --- | --- | --- | --- | --- | --- | --- | --- | --- | --- | --- | --- | --- |
|  | n (%) | *P* value | n (%) | *P* value | n (%) | *P* value | n (%) | *P* value | n (%) | *P* value | n (%) | *P* value |
| N | 690 (8) |  | 459 (5) |  | 955 (11) |  | 1987 (22) |  | 2229 (25) |  | 1882 (21) |  |
| Group |  | 0.168 |  | 0.181 |  | 0.053 |  | 0.977 |  | 0.666 |  | <0.001 |
| CML | 140 (9) |  | 95 (6) |  | 197 (12) |  | 364 (22) |  | 402 (25) |  | 421 (26) |  |
| Controls | 550 (8) |  | 364 (5) |  | 758 (10) |  | 1623 (22) |  | 1827 (25) |  | 1461 (20) |  |
| Sex |  | 0.492 |  | 0.293 |  | 0.181 |  | 0.078 |  | <0.001 |  | 0.057 |
| Male | 319 (8) |  | 207 (5) |  | 434 (10) |  | 909 (22) |  | 963 (23) |  | 857 (20) |  |
| Female | 371 (8) |  | 252 (5) |  | 521 (11) |  | 1078 (23) |  | 1266 (27) |  | 1025 (22) |  |
| Age, y, median (range) | 30 (16-81) | <0.001 | 32 (16-86) | 0.345 | 33 (16-92) | <0.001 | 33 (16-92) | <0.001 | 32 (16-92) | <0.001 | 32 (16-92) | <0.001 |
| Household registration |  | 0.445 |  | 0.138 |  | 0.038 |  | 0.354 |  | 0.048 |  | 0.072 |
| Urban | 393 (8) |  | 283 (6) |  | 587 (11) |  | 1177 (23) |  | 1340 (26) |  | 1132 (22) |  |
| Rural | 297 (8) |  | 176 (5) |  | 368 (10) |  | 810 (22) |  | 889 (24) |  | 750 (20) |  |
| Marital status |  | <0.001 |  | 0.002 |  | <0.001 |  | <0.001 |  | 0.005 |  | 0.019 |
| Unmarried | 335 (9) |  | 179 (5) |  | 335 (9) |  | 735 (20) |  | 857 (23) |  | 740 (20) |  |
| Married | 315 (6) |  | 256 (5) |  | 578 (12) |  | 1190 (24) |  | 1301 (26) |  | 1077 (22) |  |
| Divorced or widowed | 40 (17) |  | 24 (10) |  | 42 (18) |  | 62 (26) |  | 71 (30) |  | 65 (27) |  |
| Education, n (%) |  | 0.132 |  | 0.284 |  | 0.004 |  | 0.114 |  | 0.488 |  | 0.034 |
| Junior middle school and below | 83 (9) |  | 54 (6) |  | 114 (13) |  | 205 (23) |  | 212 (24) |  | 200 (22) |  |
| Senior middle school | 85 (8) |  | 58 (6) |  | 131 (13) |  | 250 (25) |  | 250 (25) |  | 242 (24) |  |
| University and above | 522 (8) |  | 347 (5) |  | 710 (10) |  | 1532 (22) |  | 1767 (25) |  | 1440 (21) |  |
| Comorbidity(ies), n (%) |  | <0.001 |  | <0.001 |  | <0.001 |  | <0.001 |  | <0.001 |  | <0.001 |
| Yes | 116 (13) |  | 92 (10) |  | 159 (17) |  | 273 (30) |  | 305 (33) |  | 285 (31) |  |
| No | 552 (7) |  | 348 (5) |  | 762 (10) |  | 1643 (22) |  | 1838 (24) |  | 1523 (20) |  |
| Residential location, n (%) |  | 0.016 |  | <0.001 |  | <0.001 |  | <0.001 |  | <0.001 |  | <0.001 |
| Hubei province | 89 (10) |  | 80 (9) |  | 146 (16) |  | 268 (30) |  | 321 (35) |  | 245 (27) |  |
| Elsewhere | 601 (8) |  | 379 (5) |  | 809 (10) |  | 1719 (22) |  | 1908 (24) |  | 1637 (21) |  |
| Residential location, n (%) |  | 0.170 |  | 0.242 |  | 0.990 |  | 0.917 |  | 0.261 |  | 0.563 |
| Urban | 536 (8) |  | 356 (5) |  | 761 (11) |  | 1582 (22) |  | 1795 (25) |  | 1491 (21) |  |
| Rural | 154 (9) |  | 103 (6) |  | 194 (11) |  | 405 (23) |  | 434 (24) |  | 391 (22) |  |
| Cohabitating with family or friends, n (%) |  | 0.076 |  | 0.994 |  | 0.953 |  | 0.694 |  | 0.690 |  | 0.661 |
| Yes | 588 (8) |  | 401 (5) |  | 835 (11) |  | 1731 (22) |  | 1953 (25) |  | 1650 (21) |  |
| No | 102 (9) |  | 58 (5) |  | 120 (11) |  | 256 (23) |  | 276 (25) |  | 232 (21) |  |
| Following pandemic information frequently, n (%) |  | <0.001 |  | <0.001 |  | <0.001 |  | <0.001 |  | <0.001 |  | <0.001 |
| Yes | 440 (10) |  | 323 (8) |  | 708 (17) |  | 1284 (31) |  | 1501 (36) |  | 1206 (29) |  |
| No | 250 (5) |  | 136 (3) |  | 247 (5) |  | 703 (15) |  | 728 (16) |  | 676 (15) |  |
| Sharing feelings actively, n (%) |  | 0.005 |  | 0.798 |  | 0.001 |  | <0.001 |  | <0.001 |  | 0.119 |
| Yes | 538 (7) |  | 374 (5) |  | 821 (11) |  | 1686 (23) |  | 1921 (26) |  | 1565 (22) |  |
| No | 152 (10) |  | 85 (5) |  | 134 (8) |  | 301 (19) |  | 308 (19) |  | 317 (20) |  |
| Having acute respiratory symptom, n (%) |  | <0.001 |  | <0.001 |  | <0.001 |  | <0.001 |  | <0.001 |  | <0.001 |
| Yes | 169 (21) |  | 113 (14) |  | 190 (24) |  | 295 (37) |  | 371 (46) |  | 344 (43) |  |
| No | 521 (6) |  | 346 (4) |  | 765 (10) |  | 1692 (21) |  | 1858 (23) |  | 1538 (19) |  |
